# Impact of policies restricting advertising, promotion, and sponsorship of sugar-sweetened beverages: A systematic review

**DOI:** 10.17843/rpmesp.2025.421.14023

**Published:** 2025-02-17

**Authors:** Leila Guarnieri, Lucas Perelli, Marcos Clausen, Germán Guaresti, Natalia Espinola, Andrea Graciano, Andrea Alcaraz

**Affiliations:** 1 InterAmerican Heart Foundation Argentina, Buenos Aires, Argentina. InterAmerican Heart Foundation Argentina Buenos Aires Argentina; 2 Institute for Clinical and Health Effectiveness, Department of Health Technology Assessment, Buenos Aires, Argentina. Institute for Clinical and Health Effectiveness Department of Health Technology Assessment Buenos Aires Argentina; 3 Dr. Ramón Carrillo Regional Hospital, Bariloche, Argentina. Dr. Ramón Carrillo Regional Hospital Bariloche Argentina; 4 National University of Río Negro, Andean Campus, Bariloche, Argentina. National University of Río Negro Andean Campus Bariloche Argentina; 5 University of Buenos Aires, Buenos Aires, Argentina. University of Buenos Aires Buenos Aires Argentina

**Keywords:** Sugar-Sweetened Beverages, Advertising, Health policy, Noncommunicable Diseases

## Abstract

**Objectives.:**

To summarize the evidence on the impact of the implementation of the ban on the advertising, promotion and sponsorship (APS) of sugar-sweetened beverages (SSBs) in terms of decreased consumption, advertising exposure and relevant clinical outcomes.

**Materials and methods.:**

Systematic review of articles published between 2001-2021 in the PubMed, Embase, Global Health, CINAHL and LILACS databases written in English, Portuguese or Spanish. We included experimental, observational and economic model studies. Risk of bias was assessed using RoB2, Quality Assessment Tool for Observational Cohort and Cross-Sectional Studies, Quality Assessment Tool for Before-After (Pre-Post) Studies With No Control Group and Consolidated Health Economic Evaluation Reporting Standards 2022. We carried out a descriptive synthesis of the studies.

**Results.:**

We selected 11 out of 1146 identified studies. Due to the heterogeneity of the outcomes, it was not possible to conduct a meta-analysis. The interventions corresponded to a comprehensive policy; restrictions on television advertising, promotions, point-of-sale advertising and advertising in schools. We found changes in clinical outcomes (obesity, cardiovascular disease, diabetes, cancer), economic outcomes (purchase, sale, cost-effectiveness, other economic outcomes), exposure and consumption. Most of the effect measures decreased as a result of the interventions. More studies on effectively implemented policies are still needed. The results of the included studies should be interpreted taking into account their methodological limitations.

**Conclusions.:**

Policies to restrict the APS of SSBs may be effective, particularly in reducing their consumption in children and adolescents, with a positive impact on their health.

## INTRODUCTION

Noncommunicable diseases (NCD), such as cardiovascular diseases, diabetes, and certain types of cancer, are the leading cause of death worldwide, affecting all age groups and all countries ^1^. The percentage of deaths attributable to NCDs increased from 60.8% in 2000 to 73.6% in 2019 worldwide and, particularly, from 77.2% to 81.3% in the Region of the Americas ^2^. NCDs also have a significant attributable cost to health systems and society in general, and this enormous disease and economic burden represents a significant barrier to achieving the Sustainable Development Goals (SDGs) ^3^.

Overweight and obesity are major risk factors for NCDs. Statistics are alarming for both adults and children and adolescents worldwide ^4^. The prevalence of obesity in children, girls and adolescents (BGaA) between the ages of 5 and 19 worldwide increased from 2.9% (95% CI: 2.6 to 3.2) in 2000 to 4.9% (95% CI: 4.6 to 5.3) in 2010, compared to 6.8% (95% CI: 6.1 to 7.6) in 2016. There have also been increases in the adult population regarding obesity rates in recent decades, reaching 13.1% (95% CI: 12.4 to 13.9) of this age group in 2016 ^2^.

The increase in overweight and obesity levels has been linked to changes in food consumption patterns, as a result of stimuli that favor the consumption of high-calorie, low-nutritional-value products ^5^. In food environments, marketing and advertising influence both preferences and food purchasing and consumption decisions, especially among children and adolescents ^6^^,^^7^. Advertising and other forms of food and beverage marketing aimed at children and adolescents are widespread and focus mainly on products with excessive fat, sugar, or sodium content, such as SD. Evidence shows that children up to the age of 11 are not mature enough to differentiate advertising content from other types of messages ^9^, which makes this population particularly vulnerable. For this reason, international organizations such as PAHO/WHO and UNICEF are calling on countries to take immediate and urgent action to address the many nuances of this issue, such as regulating the advertising, promotion, and sponsorship (APS) of unhealthy products ^5^^,^^10^^-^^12^.

The consumption of SD is an important source of calories, generally without providing any nutrients other than sugars, increasing the risk of obesity, diabetes, heart disease, cerebrovascular disease, musculoskeletal disease, kidney failure, dementia, asthma, various types of cancer, and tooth decay ^13^^,^^14^. In addition, both obesity and tooth decay can lead to other social problems such as discrimination or lack of job opportunities ^15^.

In this context, restricting APS for unhealthy food and beverage products, especially those targeting children and adolescents, aims to reduce consumption of such products and is a cost-effective, feasible measure that is generally accepted by governments, policymakers, and the public ^7^. Systematic evidence on the health impact of implementing such regulations is essential to promote effective policies. Given that there no studies that have focused specifically on measures to restrict advertising of SD, this study conducted a systematic search with the aim of summarizing the evidence on the impact of implementing a ban on APS of SD in terms of reduced consumption, advertising exposure, and relevant clinical outcomes.

**KEY MESSAGES**

Motivation for the study. Restrictions on advertising, promotion, and sponsorship (APS) of unhealthy food and beverage products aim to reduce their consumption and protect public health. No reviews have yet evaluated the impact of restricting APS of sugary drinks (SD).

Main findings. We found that comprehensive policies that include TV advertising restrictions, as well as restrictions at points of sale and in schools, can effectively reduce SD consumption, especially among children and adolescents, decrease obesity, cardiovascular disease, diabetes, and cancer, and generate economic benefits.

Implications. Evidence on the health impact of such interventions is essential to promote effective measures.

## MATERIALS AND METHODS

### Study design

We conducted a systematic review of the published literature following the PRISMA guidelines ^16^. (Supplementary material, Appendix 1).

### Search strategy

The search included studies conducted at the national, regional, and international levels indexed in the PubMed, Embase, Global Health, CINAHL, and LILACS (Latin American and Caribbean Health Science Literature) bibliographic databases. The complete search strategy can be found in the supplementary material (Appendix 2).

### Selection criteria

We included studies published in the last 20 years (2001-2021) in English, Portuguese, or Spanish; describing policies regulating APS of SD, whether mandatory or voluntary, independently or complementary to other policies; studies measuring the impact on consumption, purchases, sales, purchase intention, exposure, and/or clinical outcomes (obesity, caries, cardiovascular disease, high blood pressure, dyslipidemia, insulin resistance, diabetes, cancer, and other related clinical outcomes); and studies with experimental designs, controlled before-and-after studies, uncontrolled before-and-after studies, quasi-experimental studies, cross-sectional studies, economic models, economic evaluations, and cost studies. Studies that did not meet the inclusion criteria and those that analyzed the impact of regulatory policies on APS for non-sugar-sweetened beverages were excluded.

### Study selection and data collection

Each of the identified studies was evaluated by two reviewers from the research team assigned randomly at each stage of the process, who initially selected those that met the inclusion criteria based on the title and abstract, and subsequently by reading the full texts, using the COVIDENCE® computer program ^17^. When discrepancies arose between reviewers, they were discussed among the entire research team to reach a final consensus decision. Each of the selected studies was then randomly assigned to a researcher to identify and extract relevant information, with individual concerns that arose during the process being resolved as a group to reach a conclusion.

We constructed a descriptive summary of the main characteristics of the studies, considering the type of design, the interventions, their degree of implementation, and the outcomes. It was not possible to perform a meta-analysis given the high heterogeneity of the interventions, the target populations, and the outcomes evaluated in each study.

### Assessment of risk of bias in included studies (quality)

Pairs of reviewers independently assessed the risk of bias (quality) of the included studies. In case of disagreement, it was resolved by group consensus. Due to the nature of the research question, a single tool was not considered applicable; instead, a combination of validated instruments was used according to the study designs. The RoB2 tool ^18^ was used for randomized clinical trials, the Quality Assessment Tool for Observational Cohort and Cross-Sectional Studies ^19^ was used for cross-sectional designs, the Quality Assessment Tool for Before-After (Pre-Post) Studies With No Control Group ^19^ was used for controlled and uncontrolled before-after studies and for quasi-experimental studies; and the Consolidated Health Economic Evaluation Reporting Standards 2022 checklist ^20^ was used for economic models, developed with the main purpose of serving as a report guide.

## RESULTS

### Study selection

**Figure 1:**
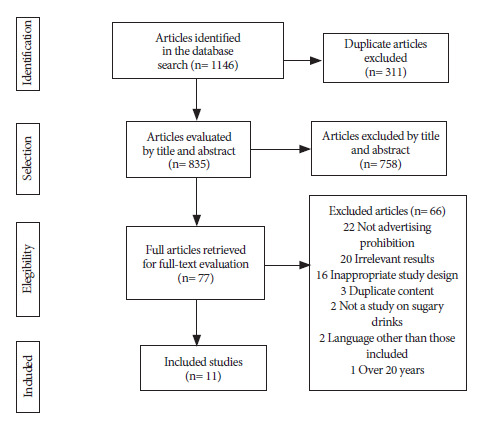
Selection process for studies included in the review.

We identified 1,146 studies from the databases using the search strategy. After removing duplicates (n=311), the remaining 835 were analyzed using the inclusion criteria based on title and abstract. A total of 758 studies were excluded in this first stage, and the remaining 77 were identified as eligible for full-text analysis. Finally, 11 articles ^(21-31)^ were eligible for this systematic review (Table 1). The complete study selection process is shown in Figure 1.

**Table 1:** Description of the included studies.

Authors	Year	Country	Design	Evaluated intervention	Analyzed variables									
					Obesity	Cardiovascular disease	Diabetes	Cancer	Purchase	Sale	Cost-effectiveness	Other economic outcomes	Consumption	Exposure
Brimblecombe *et al.*	2020	Australia	Randomized clinical trial	Restrictions on POS						x				
Brown *et al.*	2018	Australia	Economic model	Restrictions on TV advertising aimed at BGaA	x						x		x	
Correa *et al.*	2020	Chile	Uncontrolled before and after study	Restrictions on TV advertising aimed at BGaA										x
Huse *et al.*	2020	Australia	Model	Promotion restrictions	x	x	x	x			x	x	x	
Magnus *et al.*	2009	Australia	Model	Restrictions on TV advertising	x						x			
Miller *et al.*	2016	United States of America	Cross-sectional	Restriction on promotions in schools									x	
Mytton *et al.*	2020	United Kingdom	Model	Restrictions on TV advertising	x						x	x	x	x
Pauzé & Potvin Kent	2021	Canada	Quasi-experimental study	Restriction of TV advertising aimed at BGaA (self-regulation)										x
Polacsek *et al.*	2012	United States of America	Cross-sectional	Restrictions on advertising in schools										x
Potvin Kent & Wanless	2014	Canada	Uncontrolled before and after study	Restriction of TV advertising aimed at BGaA (self-regulation)										x
Taillie *et al.*	2020	Chile	Controlled before and after study	Comprehensive policy ^a^					x					

POS: Point of sale; BGaA: boys, girls and adolescents; TV: Television; ^a^ Includes front-of-pack labeling, restrictions on advertising aimed at BGaA, and restrictions on sales in schools.

### General characteristics

Regarding methodological designs, there was considerable heterogeneity among the studies, with most being economic impact models. In terms of interventions, of the eleven articles, six analyzed television advertising restrictions ^22^^,^^23^^,^^25^^,^^27^^,^^28^^,^^30^, two analyzed restrictions in schools ^(26, 29)^, one analyzed restrictions on promotions (defined as temporary price reductions and product offers) ^24^, one on point-of-sale restrictions ^21^, and one on comprehensive policy ^31^^)^ (Table 1). The supplementary material (Appendix 3) provides more details on the characteristics of each of the interventions evaluated in the studies.

Most studies evaluated exposure (n=5) ^23^^,^^27^^-^^30^, followed by obesity (n=4) ^22^^,^^24^^,^^25^^,^^27^, cost-effectiveness (n=4) ^22^^,^^24^^,^^25^^,^^27^, consumption (n=4) ^22^^,^^24^^,^^26^^,^^27^, other economic outcomes (n=2) ^24^^,^^27^, cardiovascular disease (n=1) ^24^, diabetes (n=1) ^24^, cancer (n=1) ^24^, purchase (n=1) ^31^, and sale (n=1) ^21^. No study evaluated the impact on caries, high blood pressure, dyslipidemia, or insulin resistance (Table 1).

### Assessment of evidence quality (risk of bias)

Five studies used evidence of high quality ^(22, 24, 25, 27, 31)^, three used moderate evidence ^(21, 23, 26)^, and three used low-quality evidence ^28^^-^^30^. (Table 2). The tables corresponding to the assessment of the quality of the studies according to each design are presented in the supplementary material (Appendix 4).

**Table 2:** Quality of studies included in the systematic review.

Author and year	Study design	Tool used	Result
Brimblecombe *et al*, 2020	Randomized clinical trial	RoB2	Moderate
Brown *et al*, 2018	Economic model	CHEERS 2022	High
Correa *et al*, 2020	Uncontrolled before and after study	Quality Assessment Tool for Before-After (Pre-Post) Studies With No Control Group (NIH)	Moderate
Huse *et al*, 2019	Model	CHEERS 2022	High
Magnus *et al,* 2009	Model	CHEERS 2022	High
Miller *et al*, 2016	Cross-sectional	Quality Assessment Tool for Observational Cohort and Cross-Sectional Studies (NIH)	Moderate
Mytton *et al*, 2020	Model	CHEERS 2022	High
Pauzé & Potvin Kent, 2021	Quasi-experimental study	Quality Assessment Tool for Before-After (Pre-Post) Studies With No Control Group (NIH)	Low
Polacsek *et al*, 2012	Cross-sectional	Quality Assessment Tool for Observational Cohort and Cross-Sectional Studies (NIH)	Low
Potvin Kent and Wanless, 2014	Uncontrolled before and after study	Quality Assessment Tool for Before-After (Pre-Post) Studies With No Control Group (NIH)	Low
Taillie *et al*, 2020	Controlled before and after study	Quality Assessment Tool for Before-After (Pre-Post) Studies With No Control Group (NIH)	High

CHEERS: Consolidated Health Economic Evaluation Reporting Standards.

### Results according to interventions

The study that analyzed the impact of a comprehensive policy ^31^ after 18 months of implementation in Chile, which includes the adoption of front-of-package warning labels, restrictions on advertising of labeled products to children and adolescents, and a ban on their sale and advertising in schools, reported a reduction in purchases of beverages labeled with the warnings of -23.7% (-23.8% to -23.7%).

With regard to television advertising restrictions, when evaluating exposure, several studies were inconclusive: while an economic impact model ^27^ and another study on the policy implemented in Chile ^24^ showed decreases, analyses of the voluntary measure taken by the Canadian food and beverage industry reported increases in one case ^28^ and increases and decreases in another ^30^. Regarding obesity, three studies, which evaluated changes in BMI ^22^^,^^25^ and in the number of children and adolescents with obesity ^27^, found decreases in these outcome measures. Similarly, three models ^22^^,^^25^^,^^27^ demonstrated that time restrictions on advertising unhealthy products would be cost-effective. Two of the studies ^(22, 27)^ found that advertising restrictions would lead to decreases in the daily energy intake of children and adolescents. One of the studies reported cost savings attributable to the implementation of restrictions ^27^.

Regarding restrictions on promotions, the same study ^24^ reported decreases in body weight and BMI in the population, as well as new cases prevented and years of life saved from cardiovascular disease, diabetes, and cancer as a result of eliminating all price promotions on SD. The same study ^24^ demonstrated that the intervention would be cost-effective, yield cost savings, and reduce daily energy intake.

Meanwhile, when analyzing restrictions at the point of sale, a randomized clinical trial ^21^ conducted over 12 weeks showed that following the implementation of a series of measures in retail stores in Australian towns, sales of free sugars in APSs fell by 6.8% (95% CI: -10.9% to -2.6%).

Finally, studies on restrictions in schools conducted in the United States showed, in one case ^26^, that school districts that apply restrictions on SDs have lower regular consumption of soft drinks; while another study showed that, despite a state ban on unhealthy product brands, there were still a large number of advertising strategies in the school environment, many of them involving SD ^29^.

None of the studies analyzed global APS restrictions that apply exclusively to SDs (Table 3, Annex 3).

**Table 3:** Impact identified according to types of interventions evaluated.

Evaluated intervention	Outcome	Authors and year	Main results
Comprehensive policy ^a^	Purchase	Taillie *et al*, 2020	The average daily per capita volume (mL) of “high-sugar” beverages purchased decreased by 23.7% (from −23.8% to −23.7%).
TV advertising restrictions	Obesity	Brown *et al*, 2018	The average BMI in children aged 5 to 15 years (kg/m^2^) decreased by 0.352.
		Mytton *et al*, 2020	Following the intervention, the number of children aged 5 to 17 with obesity could decrease by 4.6% (95% CI: 1.4%-9.5%), equivalent to 40,000 fewer children with obesity.
		Magnus *et al*, 2009	The restrictions would result in a reduction in BMI (units) of 0.04 (95% CI: 0.01, 0.08).
	Cost-effectiveness	Mytton *et al*, 2020	Restrictions on advertising foods and beverages high in fat, sugar, and salt would prevent 240,000 (95% CI: 65,000-530,000) DALYs, resulting in a monetary benefit of £7.4 billion (95% CI: £2 billion-£16 billion).
		Magnus *et al*, 2009	The cost-effectiveness of the intervention, measured as ICER, was AUD$ 3.70 (95% CI: $2.40-$7.70) per DALY. The intervention was considered “dominant” because it resulted in a benefit in health and a reduction in costs.
		Brown *et al*, 2018	The cost-effectiveness of the intervention, measured as ICER, showed a 100% probability of being “dominant,” resulting in 88,396 HALYs saved (95% CI: 54,559-123,199) and total cost savings of AUD$777.9 million (95% CI: AUD$369.8 million-AUD$1.2 billion) at the population level over a lifetime.
	Other economic outcomes	Mytton *et al*, 2020	The intervention would result in: Healthcare cost savings (millions): £84 (£23-£190) Social care cost savings (millions): £210 (£56-£490) Net monetary health-related benefit (millions): £7,400 (£2,000-£16,000)
	Exposure	Mytton *et al*, 2020	The intervention would result in an average reduction in exposure (number of advertisements for products high in sugar, fat, and/or sodium seen per day) in BGaA of 1.5.
		Correa *et al*, 2020	As a result of the policy implementation, there was a decrease in TV advertisements of: Number of appearances of “soft drinks”: 51% (p < 0.01) Number of appearances of “sports and energy drinks”: 23% (p < 0.01)
		Pauzé & Potvin Kent, 2021	BGaA exposure to sugary drink advertisements (measured as the number of advertisements viewed by BGaA) increased by 495% between May 2011 and May 2019.
		Potvin Kent & Wanless, 2014	Exposure, measured as the average number of advertisements for sugary drinks seen by children aged 2-11, showed differences between 2006 and 2009: juice advertisements decreased by 62.6% in Toronto and 51.6% in Vancouver; soft drink advertisements decreased by 37.8% in Toronto and increased by 11.1% in Vancouver.
	Consumption	Mytton *et al*, 2020	In response to the restrictions, there would be a reduction in the average daily energy intake in BGaA of 9.1 kcal/day (95% CI: 0.5-17.7).
		Brown *et al*, 2018	The implementation of the restriction would result in an average decrease in energy intake in children and adolescents aged 5 to 15 years of 115 kJ/day (27.5 kcal).
Promotion restrictions	Obesity	Huse *et al*, 2019	The intervention resulted in a mean change in body weight in the population (kg) of −0.11 (95% CI: −0.14 to −0.08) and a mean change in BMI in the population (kg/m2) of −0.04 (95% CI: −0.05 to −0.03).
	Cardiovascular disease	Huse *et al*, 2019	As a result of the intervention, 3,609 (95% CI: 2,625-4,688) new cases of heart disease would be prevented, saving 11,941 (95% CI: 8,967-15,322) years of life.
	Diabetes	Huse *et al*, 2019	As a result of the intervention, 14,319 (95% CI: 10,198-19,282) new cases of diabetes would be prevented and, as a consequence, 5,041 (95% CI: 3,604-6,779) years of life would be saved.
	Cancer	Huse *et al*, 2019	As a result of the intervention, 846 (95% CI: 395-1485) cases of colorectal, breast, endometrial, and kidney cancer would be prevented, saving 2,798 (95% CI: 1,822-4,067) years of life.
	Cost-effectiveness	Huse *et al*, 2019	The cost-effectiveness of the intervention, measured as ICER, was found to be dominant, with a total of 34,260 HALYs gained (24,922-45,504) and total cost savings of AUD$358.9 million (95% CI: −AUD$260.1 million to −AUD$477.7 million).
	Other economic outcomes	Huse *et al*, 2019	The intervention would result in total cost savings of: −AUD376.0 million (95% CI: −AUD277.4 million to −AUD494.3 million)
	Consumption	Huse *et al,* 2019	The intervention resulted in an average change in daily energy intake of −12.52 kJ (95% CI: −15.91 to −9.58) per person.
POS restrictions	Sale	Brimblecombe *et al*, 2020	The implementation of restrictions led to a statistically significant reduction in sales of free sugars in SD (g/total MJ): -6.8% (-10.9 to -2.6).
Restrictions in schools	Consumption	Miller *et al*, 2016	Districts that apply restrictions on promotional products have 16% less regular consumption of soft drinks.
	Exposure	Polacsek *et al*, 2012	An average of 49 food and beverage posters and signs were found in different areas, 45% of vending machine advertising was for Coca-Cola® and PepsiCo®.

BGaA: boys, girls and adolescents; DALYs: Disability-adjusted life years; ICER: Incremental cost-effectiveness ratio; HALYS: Total health-adjusted life years; POS: Point of sale; SB: Sugar-sweetened beverages; ^a^ Includes front-of-package labeling, restrictions on advertising aimed at children and adolescents, and restrictions on sales in schools. £: Pound.

## DISCUSSION

The results of this study show that interventions restricting the APS of food and beverage products with excessive amounts of sugars, fats, and/or sodium, such as SDs, could be beneficial in terms of clinical, economic, consumption, and exposure outcomes. These results are consistent with those reported by Boyland *et al*. ^32^, who found that restrictions on the marketing of unhealthy products can help reduce their purchase, their negative health consequences, and also limit exposure to and/or the power of such marketing.

One of our main findings shows that, when implementing a comprehensive policy of front-of-package warning labels, restrictions on advertising labeled products to children and adolescents, and a ban on their sale and advertising in schools in Chile, the purchase of SD decreased, proving to be an effective measure for improving health ^31^. It should be noted that in Chile, beverages sweetened with non-caloric sweeteners are not covered by the restrictions, and decreases in SD purchases were accompanied by an increase in purchases of beverages with sweeteners ^33^. In this regard, it will be essential to monitor the impact of similar policies adopted in countries such as Mexico ^34^ and Argentina ^35^, where products with non-caloric sweeteners are covered.

Furthermore, it should be noted that, worldwide, most of the implemented policies correspond to restrictions on television advertising aimed at children and adolescents ^36^; accordingly, most of the studies included in this paper refer to television restrictions, and they particularly highlight the impact of these measures on exposure to advertising for unhealthy products. However, given the increasing exposure of children and adolescents to advertising in video games and social media such as YouTube, TikTok, and Instagram ^37^, international organizations recommend that regulations also include digital media ^38^. In this regard, although some countries, such as Argentina, Chile, and the United Kingdom, have adopted measures that incorporate this type of media ^35^^,^^36^, their monitoring and enforcement by governments is recognized as one of the main challenges to be overcome ^38^.

Another noteworthy finding is the ineffectiveness of voluntary initiatives by the food and beverage industry to reduce children and adolescents’ exposure to SD advertising. Contrary to their intended objective, two evaluations included in this study show that since their implementation, their indicators increased ^28^^,^^30^. These results are consistent with those reported by Théodore *et al*., who demonstrated that, despite the self-regulation initiative in Mexico, SD companies continued to implement advertising strategies aimed at children ^39^. In this regard, it is important to note that the World Health Organization recommends that policies aimed at restricting advertising of unhealthy foods and beverages to children and adolescents be mandatory ^7^.

Furthermore, this study shows that even when restrictions are targeted at the point of sale, including strategies such as no promotional activities and no discretionary foods and beverages available at the counter ^21^, results that contribute to improving health can be achieved. In this regard, the World Health Organization has recognized retailers as a central link in the food environment, highlighting the importance of interventions in these areas ^40^, so that point-of-sale restrictions tailored to each particular context could also be effective strategies.

One of the main limitations of this study is that a significant proportion of the included studies correspond to economic impact models ^22^^,^^24^^,^^25^^,^^27^, which means that the actual impact of implementing the interventions analyzed may differ from what was reported. In addition, several studies were of low quality ^(28-30)^, and their results should therefore be interpreted with caution. In turn, the quasi-experimental study ^28^^)^ and the uncontrolled before-and-after studies ^23^^,^^30^^)^ could have biases, particularly regarding sample selection regarding the analyzed periods. Furthermore, due to the heterogeneity of the results found in the many studies, it was not possible to group them, making comparison difficult.

On the other hand, a notable limitation is the lack of evidence on policies implemented to restrict APSs in SD. It is also worth noting the scarcity of studies that report on the impact in relation to clinical outcomes, such as diabetes and cardiovascular disease. Despite this, the economic impact models included in this review provide evidence of the potential effectiveness of these measures, helping to fill these gaps. In addition, some studies ^22^^,^^27^ report outcomes from APS restrictions on products high in sugar, fat, and salt, without focusing exclusively on SD. In any case, given that SD are among the main ultra-processed products advertised ^41^ and that they provide half of the sugars in the diet ^42^, a large part of the impact of measures restricting the APS of products high in critical nutrients in general could be attributed to restrictions on SD.

The main strength of this study lies in the fact that it is one of the few systematic reviews available on the impact of APS restrictions on unhealthy food and beverage products, such as SDs. Our results demonstrate the effectiveness of different interventions across different variables and could be used to support the promotion of effective policies that contribute to improving people’s health, with a special emphasis on children and adolescents.

In conclusion, this study shows that policies that include restrictions on the APS of SD could be effective in reducing their consumption, which could lead to health benefits, especially for children and adolescents. However, it is necessary to continue working on medium- and long-term impact assessments of the measures that are beginning to be implemented in different countries around the world in order to obtain information about their effectiveness on consumption and clinical indicators such as obesity, cardiovascular disease, diabetes, and cancer. In addition, further research is needed to demonstrate the impact of the APS of SD restriction policies that extend to digital media, as well as the application of methodologies to help monitor them adequately

## References

[1] OMS (2023). Enfermedades no transmisibles.

[2] World Health Organization (2022). World health statistics 2022: monitoring health for the SDGs, sustainable development goals.

[3] World Health Organization GLOBAL STATUS REPORT on noncommunicable diseases 2014.

[4] Organización Mundial de la Salud (2024). Obesidad y sobrepeso.

[5] UNICEF Obesidad: una cuestión de derechos de niños, niñas y adolescentes. Recomendaciones de políticas para su protección.

[6] Smith R, Kelly B, Yeatman H, Boyland E (2019). Food Marketing Influences Children's Attitudes, Preferences and Consumption: A Systematic Critical Review. Nutrients.

[7] World Health Organization (2023). Policies to protect children from the harmful impact of food marketing: WHO guideline.

[8] Organización Panamericana de la Salud (2022). Consumo de productos alimentarios ultraprocesados y procesados con exceso de nutrientes asociados a las enfermedades crónicas no transmisibles y a la alimentación insalubre en las Américas.

[9] Roedder John D (1999). Consumer Socialization of Children: A Retrospective Look At Twenty Five Years of Research. Journal of Consumer Research.

[10] World Health Organization (2010). Set of recommendations on the marketing of foods and non-alcoholic beverages to children.

[11] Pan American Health Organization (2022). Regulación de la publicidad de productos alimentarios en las Américas. Casos de estudio en Brasil, Chile, México y Perú.

[12] Organización Mundial de la Salud (2016). Informe de la Comisión para acabar con la obesidad infantil.

[13] Valenzuela MJ, Waterhouse B, Aggarwal VR, Bloor K, Doran T (2021). Effect of sugar-sweetened beverages on oral health a systematic review and meta-analysis. Eur J Public Health.

[14] Malik VS, Hu FB (2022). The role of sugar-sweetened beverages in the global epidemics of obesity and chronic diseases. Nat Rev Endocrinol.

[15] Kovalskys I, Rausch HC, Indart RP, Añez EV, Zonis LN, Orellana L (2016). Childhood Obesity and Bullying in Schools of Argentina: Analysis of This Behaviour in a Context of High Prevalence. Journal of Childhood Obesity.

[16] Page MJ, Moher D, Bossuyt PM, Boutron I, Hoffmann TC, Mulrow CD (2021). PRISMA 2020 explanation and elaboration updated guidance and exemplars for reporting systematic reviews. BMJ.

[17] Veritas Health Innovation M Australia (2020). Covidence systematic review software.

[18] Sterne JAC, Savovic J, Page MJ, Elbers RG, Blencowe NS, Boutron I (2019). RoB 2 a revised tool for assessing risk of bias in randomised trials. BMJ.

[19] Study Quality Assessment Tools.

[20] Husereau D, Drummond M, Augustovski F, de Bekker-Grob E, Briggs AH, Carswell C (2022). Consolidated Health Economic Evaluation Reporting Standards (CHEERS) 2022 Explanation and Elaboration A Report of the ISPOR CHEERS II Good Practices Task Force. Value Health.

[21] Brimblecombe J, McMahon E, Ferguson M, De Silva K, Peeters A, Miles E (2020). Effect of restricted retail merchandising of discretionary food and beverages on population diet a pragmatic randomised controlled trial. Lancet Planet Health.

[22] Brown V, Ananthapavan J, Veerman L, Sacks G, Lal A, Peeters A (2018). The Potential Cost-Effectiveness and Equity Impacts of Restricting Television Advertising of Unhealthy Food and Beverages to Australian Children. Nutrients.

[23] Correa T, Reyes M, Taillie LS, Corvalán C, Dillman Carpentier FR (2020). Food Advertising on Television Before and After a National Unhealthy Food Marketing Regulation in Chile, 2016-2017. Am J Public Health.

[24] Huse O, Ananthapavan J, Sacks G, Cameron AJ, Zorbas C, Peeters A (2020). The potential cost-effectiveness of mandatory restrictions on price promotions for sugar-sweetened beverages in Australia. Int J Obes.

[25] Magnus A, Haby MM, Carter R, Swinburn B (2009). The cost-effectiveness of removing television advertising of high-fat and/or high-sugar food and beverages to Australian children. Int J Obes.

[26] Miller GF, Sliwa S, Brener ND, Park S, Merlo CL (2016). School District Policies and Adolescents' Soda Consumption. J Adolesc Health.

[27] Mytton OT, Boyland E, Adams J, Collins B, O'Connell M, Russell SJ (2020). The potential health impact of restricting less-healthy food and beverage advertising on UK television between 05 30 and 21.00 hours: A modelling study. PLoS Med.

[28] Pauzé E, Potvin Kent MP (2021). Children's measured exposure to food and beverage advertising on television in Toronto (Canada), May 2011-May 2019. Canadian Journal of Public Health.

[29] Polacsek M, O'Rourke K, O'Brien L, Blum JW, Donahue S (2012). Examining compliance with a statewide law banning junk food and beverage marketing in Maine schools. Public Health Rep.

[30] Potvin Kent M, Wanless A (2014). The influence of the Children's Food and Beverage Advertising Initiative change in children's exposure to food advertising on television in Canada between 2006-2009. International Journal of Obesity.

[31] Taillie LS, Reyes M, Colchero MA, Popkin B, Corvalán C (2020). An evaluation of Chile's Law of Food Labeling and Advertising on sugar-sweetened beverage purchases from 2015 to 2017: A before-and-after study. PLoS Med.

[32] Boyland E, McGale L, Maden M, Hounsome J, Boland A, Jones A (2022). Systematic review of the effect of policies to restrict the marketing of foods and non-alcoholic beverages to which children are exposed. Obes Rev.

[33] Rebolledo N, Bercholz M, Adair L, Corvalán C, Ng SW, Taillie LS (2022). Sweetener purchases in Chile before and after implementing a policy for food labeling, marketing, and sales in schools. Current Developments in Nutrition.

[34] Ministerio de Economía de México (2020). Modificación a la Norma Oficial Mexicana NOM-051-SCFI/SSA1-2010, Especificaciones generales de etiquetado para alimentos y bebidas no alcohólicas preenvasados-Información comercial y sanitaria, publicada el 5 de abril de 2010.

[35] Boletin Oficial de la República Argentina (2021). Promoción de la Alimentación Saludable. Ley 27642.

[36] Taillie LS, Busey E, Stoltze FM, Dillman Carpentier FR (2019). Governmental policies to reduce unhealthy food marketing to children. Nutr Rev.

[37] UNICEF Argentina (2021). Exposición de niños, niñas y adolescentes al marketing digital de alimentos y bebidas en Argentina.

[38] Monitoring and restricting digital marketing of unhealthy products to children and adolescents (2018). Report based on the expert meeting on monitoring of digital marketing of unhealthy products to children and adolescents.

[39] Théodore FL, Tolentino-Mayo L, Hernández-Zenil E, Bahena L, Velasco A, Popkin B (2017). Pitfalls of the self-regulation of advertisements directed at children on Mexican television. Pediatr Obes.

[40] World Health Organization, Regional Office for Europe (2022). WHO European regional obesity report 2022.

[41] Kumar G, Onufrak S, Zytnick D, Kingsley B, Park S (2015). Self-reported advertising exposure to sugar-sweetened beverages among US youth. Public Health Nutr.

[42] Organización Panamericana de la Salud (2019). Alimentos y bebidas ultraprocesados en América Latina: ventas, fuentes, perfiles de nutrientes e implicaciones.

