# Supplementary material for: Impact of policies restricting advertising, promotion, and sponsorship of sugar-sweetened beverages: A systematic review
Source: Rev Peru Med Exp Salud Publica. 2025 Feb 17;42(1):28–36. doi: 10.17843/rpmesp.2025.421.14023 (PMC12176025; doi:10.17843/rpmesp.2025.421.14023)
Supplement: Supplementary material. — Available in the electronic version of the RPMESP. [file rpmesp-42-01-14023-s001.docx]

**Material suplementario**

**Anexo 1:** Checklist PRISMA^(1)^

| **Sección/tema** | **ítem #** | **Checklist ítem** | **Página** |
| --- | --- | --- | --- |
| **TÍTULO** |  |  |  |
| Titulo | 1 | Identificar la publicación como revisión sistemática | 1 |
| **RESUMEN** |  |  |  |
| Resumen | 2 | Consulte la lista de verificación de PRISMA 2020 para resúmenes | 1 |
| **INTRODUCCION** |  |  |  |
| Fundamentos | 3 | Describir la justificación de la revisión en el contexto de los conocimientos existentes. | 2 |
| Objetivos | 4 | Proporcionar una declaración explícita de los objetivos o preguntas que la revisión desea contestar. | 2 |
| **MÉTODOS** |  |  |  |
| Criterios de elegibilidad | 5 | Especificar los criterios de inclusión y exclusión para la revisión y cómo se agruparon los estudios para la síntesis. | 2 y 3 |
| Fuentes de información | 6 | Especificar todas las bases de datos, registros, sitios web, organizaciones, listas de referencia y otras fuentes buscadas o consultadas para identificar estudios. Especifique la fecha en la que se buscó o consultó por última vez cada fuente. | 2 |
| Estrategia de búsqueda | 7 | Presentar las estrategias de búsqueda completas para todas las bases de datos, registros y sitios web, incluidos los filtros y los límites utilizados. | MS^[[1]](#footnote-1)^ |
| Proceso de selección | 8 | Especificar los métodos utilizados para decidir si un estudio cumplía los criterios para la inclusión de la revisión, incluidos cuántos revisores  examinaron cada registro y cada informe recuperado, si trabajaron de forma independiente y, si procede, los detalles de las herramientas de automatización utilizadas en el proceso. | 2 y 3 |
| Proceso de recopilación de datos | 9 | Especificar los métodos utilizados para recopilar los datos de los estudios, incluido el número de revisores que recopilaron datos de cada informe, si trabajaron de forma independiente, los procesos para obtener o confirmar datos de los investigadores del estudio y, si procede, los detalles de las herramientas de automatización utilizadas en el proceso. | 3 |
| Lista de datos | 10a | Enumerar y definir todos los resultados para los que se buscaron los datos. Especifique si se buscaron todos los resultados admitidos por cada dominio de resultados en cada estudio (por ejemplo, para todas las medidas, puntos de tiempo, análisis) y, si no, los métodos utilizados para decidir qué resultados recopilar. | 6 |
|  | 10b | Enumerar y definir todas las demás variables para las que se solicitaron datos (por ejemplo, características de participante e intervención, fuentes  de financiación). Describa cualquier suposición hecha sobre cualquier información que falte o no esté clara. | 3 |
| Evaluación del riesgo de sesgo | 11 | Especificar los métodos utilizados para evaluar el riesgo de sesgo en los estudios incluidos, incluidos los detalles de las herramientas utilizadas, cuántos revisores evaluaron cada estudio y si trabajaron de forma independiente y, si procede, los detalles de las herramientas de automatización utilizadas en el proceso. | 3,5 y MS |
| Medidas de efecto | 12 | Especificar para cada resultado como se midió el efecto (por ejemplo, relación de riesgo, diferencia media) utilizadas en la síntesis o presentación de resultados. | 3 |
| Métodos de síntesis | 13a | Describir los procesos utilizados para decidir qué estudios eran elegibles para cada síntesis. | 3 |
|  | 13b | Describir los métodos necesarios para preparar los datos para la presentación o síntesis, como el manejo de las estadísticas de resumen que faltan o las conversiones de datos. | No aplicable |
|  | 13c | Describir cualquier método utilizado para tabular o mostrar visualmente los resultados de estudios y síntesis individuales. | No aplicable |
|  | 13d | Describir los métodos utilizados para sintetizar resultados y proporcionar justificación para las opciones. Si se realizó un metanálisis, describa los modelos, los métodos para identificar la presencia y el alcance de la heterogeneidad estadística y los paquetes de software utilizados. | 3 |
|  | 13e | Describa los métodos utilizados para explorar las posibles causas de la heterogeneidad entre los resultados del estudio | No aplicable |
|  | 13f | Describir los análisis de sensibilidad realizados para evaluar la fuerza de los resultados sintetizados. | No aplicable |
| Informar de la evaluación del sesgo | 14 | Describir cualquier método utilizado para evaluar el riesgo de sesgo debido a la falta de resultados en una síntesis (derivada de sesgos de notificación). | No aplicable |
| Evaluación de la certeza | 15 | Describir cualquier método utilizado para evaluar la certeza (o confianza) en el cuerpo de evidencia para un resultado. | No aplicable |
| **RESULTADOS** |  |  |  |
| Selección de los estudios | 16a | Describir los resultados del proceso de búsqueda y selección, desde el número de registros identificados en la búsqueda hasta el número de estudios incluidos en la revisión, idealmente utilizando un diagrama de flujo (consulte la figura 1). | 3 |
|  | 16b | Citar estudios que cumplieran muchos criterios de inclusión, pero no todos ('casi perdidos') y explicar por qué fueron excluidos. | No aplicable |
| Características del estudio | 17 | Citar cada estudio incluido y muestre sus características. | 3, 4 y 5 |
| Riesgo de sesgo en los estudios | 18 | Presentar evaluaciones del riesgo de sesgo para cada estudio incluido | 4,5 y MS |
| Resultados de estudios individuales | 19 | Para los resultados de cada estudio: a) estadísticas resumidas para cada grupo (cuando proceda) y b) una estimación de efectos y su precisión (por ejemplo, confianza/intervalo creíble), idealmente utilizando tablas o gráficas estructuradas. | 3, 4 y 5 |
| Resultados de la síntesis | 20a | Para cada combinación o síntesis, resuma brevemente las características y el riesgo de sesgo entre los estudios. | 3, 4 y 5 |
|  | 20b | Presentar los resultados de todas las combinaciones o síntesis estadísticas realizadas. Si se realizó un metanálisis, presente para cada estimación de resumen y su precisión (por ejemplo. confianza/intervalo creíble) y medidas estadísticas de heterogeneidad. Si compara grupos, describa la dirección del efecto. | No aplicable |
|  | 20c | Presentar resultados de toda la investigación de posibles causas de heterogeneidad entre los resultados del estudio. | No aplicable |
|  | 20d | Presentar los resultados de todos los análisis de sensibilidad realizados para evaluar la solidez de los resultados combinados. | No aplicable |
| Reportar sesgos | 21 | Presentar evaluaciones del riesgo de sesgo debido a la falta de resultados (derivados de sesgos de notificación) para cada combinacion evaluada. | No aplicable |
| Certeza de la evidencia | 22 | Presentar evaluaciones de certeza (o confianza) en el cuerpo de la evidencia de cada resultado evaluado. | No aplicable |
| **DISCUSIÓN** |  |  |  |
| Discusión | 23a | Proporcionar una interpretación general de los resultados en el contexto de otras pruebas. | 7 y 8 |
|  | 23b | Discuta cualquier limitación de la evidencia incluida en el examen. | 7 |
|  | 23c | Discutir las limitaciones de los procesos de revisión utilizados. | 7 |
|  |  |  |  |
|  | 23d | Discutir las implicaciones de los resultados para la práctica, la política y la investigación futura. | 8 |
| **OTRA INFORMACIÓN** |  |  |  |
| Registro y protocolo | 24a | Proporcione información del registro de la revisión, incluido el nombre del registro y el número de registro, o indique que la revisión no se registró. | NO |
|  | 24b | Indique dónde se puede acceder al protocolo de revisión o indique que no se ha preparado un protocolo. | NO |
|  | 24c | Describir y explicar cualquier cambio en la información proporcionada en el registro o protocolo. | No aplicable |
| Apoyo | 25 | Describa las fuentes de apoyo financiero o no financiero para su revisión, y el papel de los financiadores o patrocinadores en la revisión. | 8 |
| Conflicto de intereses competitivos | 26 | Declarar cualquier conflicto de interés de los autores de las revisiones. | 8 |
| Disponibilidad de datos, código y otros materiales | 27 | Informe cuáles de las siguientes opciones están disponibles públicamente y dónde se pueden encontrar: formularios de recopilación de datos de plantilla; datos extraídos de estudios incluidos; datos utilizados para todos los análisis; código analítico; cualquier otro material utilizado en la  revisión. | NO |

**Anexo 2.** Estrategia de búsqueda

PubMed 14/12/2021

| Search | Query | Results |
| --- | --- | --- |
| #54 | #33 AND #45 AND #53 | [466](https://pubmed.ncbi.nlm.nih.gov/?term=%2333+AND+%2345+AND+%2353&ac=no&sort=relevance) |
| #53 | #46 OR #47 OR #48 OR #49 OR #50 OR #51 OR #52 | [464,725](https://pubmed.ncbi.nlm.nih.gov/?term=%2346+OR+%2347+OR+%2348+OR+%2349+OR+%2350+OR+%2351+OR+%2352&ac=no&sort=relevance) |
| #52 | Promotion*[tiab] | [112,135](https://pubmed.ncbi.nlm.nih.gov/?term=Promotion%2A%5Btiab%5D&ac=no&sort=relevance) |
| #51 | Commercial*[tiab] | [291,790](https://pubmed.ncbi.nlm.nih.gov/?term=Commercial%2A%5Btiab%5D&ac=no&sort=relevance) |
| #50 | Publicit*[tiab] | [3,260](https://pubmed.ncbi.nlm.nih.gov/?term=Publicit%2A%5Btiab%5D&ac=no&sort=relevance) |
| #49 | Advertis*[tiab] | [19,746](https://pubmed.ncbi.nlm.nih.gov/?term=Advertis%2A%5Btiab%5D&ac=no&sort=relevance) |
| #48 | Telemarketing[tiab] | [61](https://pubmed.ncbi.nlm.nih.gov/?term=Telemarketing%5Btiab%5D&ac=no&sort=relevance) |
| #47 | Marketing[tiab] | [29,830](https://pubmed.ncbi.nlm.nih.gov/?term=Marketing%5Btiab%5D&ac=no&sort=relevance) |
| #46 | Marketing[Mesh] | [36,437](https://pubmed.ncbi.nlm.nih.gov/?term=Marketing%5BMesh%5D&ac=no&sort=relevance) |
| #45 | #34 OR #35 OR #36 OR #37 OR #38 OR #39 OR #40 OR #41 OR #42 OR #43 OR #44 | [2,670,379](https://pubmed.ncbi.nlm.nih.gov/?term=%2334+OR+%2335+OR+%2336+OR+%2337+OR+%2338+OR+%2339+OR+%2340+OR+%2341+OR+%2342+OR+%2343+OR+%2344&ac=no&sort=relevance) |
| #44 | Laws[tiab] | [33,091](https://pubmed.ncbi.nlm.nih.gov/?term=Laws%5Btiab%5D&ac=no&sort=relevance) |
| #43 | Law[tiab] | [98,636](https://pubmed.ncbi.nlm.nih.gov/?term=Law%5Btiab%5D&ac=no&sort=relevance) |
| #42 | Regulat*[tiab] | [2,057,890](https://pubmed.ncbi.nlm.nih.gov/?term=Regulat%2A%5Btiab%5D&ac=no&sort=relevance) |
| #41 | Jurisprudence*[tiab] | [6,796](https://pubmed.ncbi.nlm.nih.gov/?term=Jurisprudence%2A%5Btiab%5D&ac=no&sort=relevance) |
| #40 | Legislat*[tiab] | [53,878](https://pubmed.ncbi.nlm.nih.gov/?term=Legislat%2A%5Btiab%5D&ac=no&sort=relevance) |
| #39 | Restrict*[tiab] | [494,693](https://pubmed.ncbi.nlm.nih.gov/?term=Restrict%2A%5Btiab%5D&ac=no&sort=relevance) |
| #38 | Banned[tiab] | [4,665](https://pubmed.ncbi.nlm.nih.gov/?term=Banned%5Btiab%5D&ac=no&sort=relevance) |
| #37 | Bans[tiab] | [2,687](https://pubmed.ncbi.nlm.nih.gov/?term=Bans%5Btiab%5D&ac=no&sort=relevance) |
| #36 | Ban[tiab] | [9,555](https://pubmed.ncbi.nlm.nih.gov/?term=Ban%5Btiab%5D&ac=no&sort=relevance) |
| #35 | Prohibit*[tiab] | [25,335](https://pubmed.ncbi.nlm.nih.gov/?term=Prohibit%2A%5Btiab%5D&ac=no&sort=relevance) |
| #34 | Legislation, Food[Mesh] | [2,475](https://pubmed.ncbi.nlm.nih.gov/?term=Legislation%2C+Food%5BMesh%5D&ac=no&sort=relevance) |
| #33 | #22 OR #32 | [60,281](https://pubmed.ncbi.nlm.nih.gov/?term=%2322+OR+%2332&ac=no&sort=relevance) |
| #32 | #28 AND #31 | [5,948](https://pubmed.ncbi.nlm.nih.gov/?term=%2328+AND+%2331&ac=no&sort=relevance) |
| #31 | #29 OR #30 | [175,499](https://pubmed.ncbi.nlm.nih.gov/?term=%2329+OR+%2330&ac=no&sort=relevance) |
| #30 | Drink*[tiab] | [152,949](https://pubmed.ncbi.nlm.nih.gov/?term=Drink%2A%5Btiab%5D&ac=no&sort=relevance) |
| #29 | Beverage*[tiab] | [30,807](https://pubmed.ncbi.nlm.nih.gov/?term=Beverage%2A%5Btiab%5D&ac=no&sort=relevance) |
| #28 | #23 OR #24 OR #25 OR #26 OR #27 | [107,957](https://pubmed.ncbi.nlm.nih.gov/?term=%2323+OR+%2324+OR+%2325+OR+%2326+OR+%2327&ac=no&sort=relevance) |
| #27 | Fructose*[tiab] | [33,831](https://pubmed.ncbi.nlm.nih.gov/?term=Fructose%2A%5Btiab%5D&ac=no&sort=relevance) |
| #26 | Sucrose*[tiab] | [71,089](https://pubmed.ncbi.nlm.nih.gov/?term=Sucrose%2A%5Btiab%5D&ac=no&sort=relevance) |
| #25 | Syrupe*[tiab] | [2](https://pubmed.ncbi.nlm.nih.gov/?term=Syrupe%2A%5Btiab%5D&ac=no&sort=relevance) |
| #24 | Dietary Sugars[Mesh] | [5,531](https://pubmed.ncbi.nlm.nih.gov/?term=Dietary+Sugars%5BMesh%5D&ac=no&sort=relevance) |
| #23 | Sweetening Agents[Mesh] | [11,707](https://pubmed.ncbi.nlm.nih.gov/?term=Sweetening+Agents%5BMesh%5D&ac=no&sort=relevance) |
| #22 | #1 OR #2 OR #3 OR #4 OR #5 OR #6 OR #7 OR #8 OR #9 OR #10 OR #11 OR #12 OR #13 OR #14 OR #15 OR #16 OR #17 OR #18 OR #19 OR #20 OR #21 | [56,860](https://pubmed.ncbi.nlm.nih.gov/?term=%231+OR+%232+OR+%233+OR+%234+OR+%235+OR+%236+OR+%237+OR+%238+OR+%239+OR+%2310+OR+%2311+OR+%2312+OR+%2313+OR+%2314+OR+%2315+OR+%2316+OR+%2317+OR+%2318+OR+%2319+OR+%2320+OR+%2321&ac=no&sort=relevance) |
| #21 | Fizzy Beverage*[tiab] | [2,406](https://pubmed.ncbi.nlm.nih.gov/?term=Fizzy+Beverage%2A%5Btiab%5D&ac=no&sort=relevance) |
| #20 | Fizzy Drink*[tiab] | [90](https://pubmed.ncbi.nlm.nih.gov/?term=Fizzy+Drink%2A%5Btiab%5D&ac=no&sort=relevance) |
| #19 | Juice*[tiab] | [36,030](https://pubmed.ncbi.nlm.nih.gov/?term=Juice%2A%5Btiab%5D&ac=no&sort=relevance) |
| #18 | Fruit Beverage*[tiab] | [99](https://pubmed.ncbi.nlm.nih.gov/?term=Fruit+Beverage%2A%5Btiab%5D&ac=no&sort=relevance) |
| #17 | Fruit Drink*[tiab] | [367](https://pubmed.ncbi.nlm.nih.gov/?term=Fruit+Drink%2A%5Btiab%5D&ac=no&sort=relevance) |
| #16 | Sport Beverage*[tiab] | [11](https://pubmed.ncbi.nlm.nih.gov/?term=Sport+Beverage%2A%5Btiab%5D&ac=no&sort=relevance) |
| #15 | Sport Drink*[tiab] | [117](https://pubmed.ncbi.nlm.nih.gov/?term=Sport+Drink%2A%5Btiab%5D&ac=no&sort=relevance) |
| #14 | Cola[tiab] | [1,791](https://pubmed.ncbi.nlm.nih.gov/?term=Cola%5Btiab%5D&ac=no&sort=relevance) |
| #13 | Sugary Beverage*[tiab] | [267](https://pubmed.ncbi.nlm.nih.gov/?term=Sugary+Beverage%2A%5Btiab%5D&ac=no&sort=relevance) |
| #12 | Sugared Beverage*[tiab] | [53](https://pubmed.ncbi.nlm.nih.gov/?term=Sugared+Beverage%2A%5Btiab%5D&ac=no&sort=relevance) |
| #11 | Sugary Drink*[tiab] | [570](https://pubmed.ncbi.nlm.nih.gov/?term=Sugary+Drink%2A%5Btiab%5D&ac=no&sort=relevance) |
| #10 | Sugared Drink*[tiab] | [41](https://pubmed.ncbi.nlm.nih.gov/?term=Sugared+Drink%2A%5Btiab%5D&ac=no&sort=relevance) |
| #9 | Sweetened Beverage*[tiab] | [4,115](https://pubmed.ncbi.nlm.nih.gov/?term=Sweetened+Beverage%2A%5Btiab%5D&ac=no&sort=relevance) |
| #8 | Sweetened Drink*[tiab] | [336](https://pubmed.ncbi.nlm.nih.gov/?term=Sweetened+Drink%2A%5Btiab%5D&ac=no&sort=relevance) |
| #7 | Soft Beverage*[tiab] | [39](https://pubmed.ncbi.nlm.nih.gov/?term=Soft+Beverage%2A%5Btiab%5D&ac=no&sort=relevance) |
| #6 | Soft Drink*[tiab] | [4,186](https://pubmed.ncbi.nlm.nih.gov/?term=Soft+Drink%2A%5Btiab%5D&ac=no&sort=relevance) |
| #5 | Carbonated Drink*[tiab] | [317](https://pubmed.ncbi.nlm.nih.gov/?term=Carbonated+Drink%2A%5Btiab%5D&ac=no&sort=relevance) |
| #4 | SSB[tiab] | [5,798](https://pubmed.ncbi.nlm.nih.gov/?term=SSB%5Btiab%5D&ac=no&sort=relevance) |
| #3 | Carbonated Beverage*[tiab] | [598](https://pubmed.ncbi.nlm.nih.gov/?term=Carbonated+Beverage%2A%5Btiab%5D&ac=no&sort=relevance) |
| #2 | Soda*[tiab] | [5,637](https://pubmed.ncbi.nlm.nih.gov/?term=Soda%2A%5Btiab%5D&ac=no&sort=relevance) |
| #1 | Carbonated Beverages[Mesh] | [3,148](https://pubmed.ncbi.nlm.nih.gov/?term=Carbonated+Beverages%5BMesh%5D&ac=no&sort=relevance) |

EMBase (OVID) 15/12/2021

Embase Classic+Embase <2001 to 2021 December 14>

1         exp carbonated beverage/     3544

2         Soda*.ti,ab.   7022

3         (Carbonated adj3 Beverage*).ti,ab.    817

4         SSB.ti,ab.         7155

5         (Carbonated adj3 Drink*).ti,ab. 856

6         (Soft adj3 Drink*).ti,ab. 5452

7         (Soft adj3 Beverage*).ti,ab.    266

8         Sweetened Drink*.ti,ab.          461

9         (Sweetened adj3 Beverage*).ti,ab.     5230

10       (Sugar* adj3 Drink*).ti,ab.      2135

11       (Sugar* adj3 Beverage*).ti,ab.  5047

12       Cola.ti,ab.       2295

13       (Sport adj3 Drink*).ti,ab.         193

14       (Sport adj3 Beverage*).ti,ab. 27

15       (Fruit adj3 Drink*).ti,ab.           1151

16       (Fruit adj3 Beverage*).ti,ab.   662

17       Juice*.ti,ab.   44703

18       (Fizzy adj3 Drink*).ti,ab.           159

19       (Fizzy adj3 Beverage*).ti,ab.  11

20       or/1-19 71789

21       exp sweetening agent/ 104993

22       exp sugar intake/        9176

23       Syrupe*.ti,ab.   2

24       Sucrose*.ti,ab. 83192

25       Fructose*.ti,ab. 40961

26       or/21-25          172317

27       Beverage*.ti,ab.          38093

28       Drink*.ti,ab.   209502

29       27 or 28            236783

30       26 and 29        9233

31       20 or 30            77814

32       exp food legislation/  15

33       Prohibit*.ti,ab. 34009

34       Ban.ti,ab.        10680

35       Bans.ti,ab.      3070

36       Banned.ti,ab. 5868

37       Restrict*.ti,ab.  618981

38       Legislat*.ti,ab.  64628

39       Jurisprudence*.ti,ab. 1671

40       Regulat*.ti,ab.  2539821

41       Law.ti,ab.        113197

42       or/32-41          3265779

43       exp marketing/ 26423

44       Marketing.ti,ab.           39739

45       Telemarketing.ti,ab.  60

46       Adverti*.ti,ab.  24620

47       Publicit*.ti,ab.   4259

48       or/43-47          78337

49       31 and 42 and 48         317

Global Health (OVID) 15/12/2021

Global Health <2001 to 2021 Week 49>

1         Soda*.ti,ab.   3053

2         (Carbonated adj3 Beverage*).ti,ab.    525

3         SSB.ti,ab.         1127

4         (Carbonated adj3 Drink*).ti,ab. 571

5         (Soft adj3 Drink*).ti,ab. 3806

6         (Soft adj3 Beverage*).ti,ab.    220

7         (Sweetened adj3 Drink*).ti,ab. 694

8         (Sweetened adj3 Beverage*).ti,ab.     3266

9         (Sugar* adj3 Drink*).ti,ab.      1289

10       (Sugar* adj3 Beverage*).ti,ab.  3223

11       Cola.ti,ab.       973

12       (Sport adj3 Drink*).ti,ab.         150

13       (Sport adj3 Beverage*).ti,ab. 24

14       (Fruit adj3 Drink*).ti,ab.           1063

15       (Fruit adj3 Beverage*).ti,ab.   739

16       Juice*.ti,ab.   32467

17       (Fizzy adj3 Drink*).ti,ab.           69

18       (Fizzy adj3 Beverage*).ti,ab.  9

19       or/1-18 43942

20       Syrupe*.ti,ab.   2

21       Sucrose*.ti,ab. 25110

22       Fructose*.ti,ab. 12679

23       or/20-22          34337

24       Beverage*.ti,ab.          26905

25       Drink*.ti,ab.   92151

26       24 or 25            112564

27       23 and 26        2751

28       19 or 27            45857

29       Prohibit*.ti,ab. 6284

30       Ban.ti,ab.        3075

31       Bans.ti,ab.      1210

32       Banned.ti,ab. 2100

33       Restrict*.ti,ab.  94101

34       Legislat*.ti,ab.  20060

35       Jurisprudence*.ti,ab. 113

36       Regulat*.ti,ab.  222414

37       Law.ti,ab.        15082

38       or/29-37          341615

39       Marketing.ti,ab.           13924

40       Telemarketing.ti,ab.  14

41       Adverti*.ti,ab.  6429

42       Publicit*.ti,ab.   1741

43       or/39-42          20769

44       28 and 38 and 43         194

CINAHL (Complete-EBSCO) 15/12/2021

| **#** | **Query** | **Results** |
| --- | --- | --- |
| S47 | S28 AND S39 AND S46 | 25 |
| S46 | S40 OR S41 OR S42 OR S43 OR S44 OR S45 | 50,696 |
| S45 | TI Promotion* OR AB Promotion* | 9,414 |
| S44 | TI Commercial* OR AB Commercial* | 21,163 |
| S43 | TI Publicit* OR AB Publicit* | 540 |
| S42 | TI Advertis* OR AB Advertis* | 6,929 |
| S41 | TI Telemarketing OR AB Telemarketing | 24 |
| S40 | TI Marketing OR AB Marketing | 15,733 |
| S39 | S29 OR S30 OR S31 OR S32 OR S33 OR S34 OR S35 OR S36 OR S37 OR S38 | 150,591 |
| S38 | TI Laws OR AB Laws | 42,308 |
| S37 | TI Law OR AB Law | 42,308 |
| S36 | TI Regulat* OR AB Regulat* | 74,009 |
| S35 | TI Jurisprudence* OR AB Jurisprudence* | 760 |
| S34 | TI Legislat* OR AB Legislat* | 16,336 |
| S33 | TI Restrict* OR AB Restrict* | 32,236 |
| S32 | TI Banned OR AB Banned | 404 |
| S31 | TI Bans OR AB Bans | 2,029 |
| S30 | TI Ban OR AB Ban | 2,029 |
| S29 | TI Prohibit* OR AB Prohibit* | 3,173 |
| S28 | S19 OR S27 | 817 |
| S27 | S23 AND S26 | 5 |
| S26 | S24 OR S25 | 3,066 |
| S25 | TI Drink* OR AB Drink* | 2,132 |
| S24 | TI Beverage* OR AB Beverage* | 1,109 |
| S23 | S20 OR S21 OR S22 | 36 |
| S22 | TI Fructose* OR AB Fructose* | 26 |
| S21 | TI Sucrose* OR AB Sucrose* | 10 |
| S20 | TI Syrupe* OR AB Syrupe* | 0 |
| S19 | S1 OR S2 OR S3 OR S4 OR S5 OR S6 OR S7 OR S8 OR S9 OR S10 OR S11 OR S12 OR S13 OR S14 OR S15 OR S16 OR S17 OR S18 | 815 |
| S18 | TI (Fizzy N3 Beverage*) OR AB (Fizzy N3 Beverage*) | 0 |
| S17 | TI (Fizzy N3 Drink*) OR AB (Fizzy N3 Drink*) | 1 |
| S16 | TI Juice* OR AB Juice* | 228 |
| S15 | TI (Fruit* N3 Beverage*) OR AB (Fruit* N3 Beverage*) | 13 |
| S14 | TI (Fruit* N3 Drink*) OR AB (Fruit* N3 Drink*) | 16 |
| S13 | TI (Sport N1 Beverage*) OR AB (Sport N1 Beverage*) | 47 |
| S12 | TI (Sport N1 Drink*) OR AB (Sport N1 Drink*) | 5 |
| S11 | TI Cola OR AB Cola | 154 |
| S10 | TI (Sugar* N3 Beverage*) OR AB (Sugar* N3 Beverage*) | 109 |
| S9 | TI (Sugar* N3 Drink*) OR AB (Sugar* N3 Drink*) | 44 |
| S8 | TI (Sweetened N1 Beverage*) OR AB (Sweetened N1 Beverage*) | 103 |
| S7 | TI (Sweetened N1 Drink*) OR AB (Sweetened N1 Drink*) | 2 |
| S6 | TI (Soft N3 Beverage*) OR AB (Soft N3 Beverage*) | 5 |
| S5 | TI (Soft N3 Drink*) OR AB (Soft N3 Drink*) | 221 |
| S4 | TI (Carbonated N3 Drink*) OR AB (Carbonated N3 Drink*) | 56 |
| S3 | TI SSB OR AB SSB | 101 |
| S2 | TI (Carbonated N3 Beverage*) OR AB (Carbonated N3 Beverage*) | 12 |
| S1 | TI Soda* OR AB Soda* | 130 |

LILACS (BVS-Eng) 15/12/2021

| Database : | **LILACS** |
| --- | --- |
| Search on : | **(MH Carbonated Beverages OR Gaseosa$ OR Soda$ OR SSB OR Cola OR Juice$ OR Jugo$ OR Suco$ OR ((Carbonat$ OR Soft OR Sweetened OR Sugar$ OR Azucarad$ OR Açucarad$ OR Sport OR Energizante$ OR Deportiv$ OR Fruit OR Fizzy OR Saborizad$) AND (Drink$ OR Beverage$ OR Bebida$ OR Drinque$))) AND (MH Legislation, Food OR Prohibi$ OR Ban OR Bans OR Banned OR Restrict$ OR Restriccion$ OR Restrição OR Restringid$ OR Legisla$ OR Jurispruden$ OR Regula$ OR Law$ OR Ley$ OR Lei OR Leis OR Limitacion$ OR Limitacao$) AND (MH Marketing OR Marketing OR Telemarketing OR Advertis$ OR Publici$ OR Commercial$ OR Comercial$ OR Comerciais OR Promotion OR Promocion$ OR Promocao$) [Words]** |
| References found : | **144** [[refine](http://bases.bireme.br/cgi-bin/wxislind.exe/iah/online/#refine)] |

**Anexo 3:** Descripción de las intervenciones evaluadas

| **Autores** | **Año** | **Características de la intervención evaluada** | **Duración de la intervención** | **Ámbito de aplicación** |
| --- | --- | --- | --- | --- |
| Brimblecombe et al | 2020 | Restricción de la actividad promocional en alimentos y bebidas no esenciales; Restricción de la actividad promocional engañosa  Restricción de la disponibilidad visible de alimentos y bebidas no esenciales, reemplazándolos con productos más saludables; Reducción de los espacios de azúcar de mesa, golosinas y galletas dulces en favor de productos más saludables o productos no alimenticios; Reducción de los espacios en refrigeradores para bebidas objetivo, sustituyéndolas con bebidas más saludables; No se permiten bebidas gaseosas de más de 600 mililitros en refrigeradores; Utilización de pegatinas y etiquetas indicando el contenido de azúcar en gaseosas y promoviendo el consumo de agua | 12 semanas | Puntos de venta minoristas de localidades de Australia |
| Brown et al | 2018 | Restricción horaria en la publicidad de alimentos y bebidas no saludables dirigida a niños menores de 16 años en TV abierta hasta las 9:30 p.m | N/A | Canales de TV abierta de Australia |
| Correa et al | 2020 | Restricción de la publicidad en TV dirigida a NNyA de productos altos en energía, azúcares, grasas saturadas y sodio. Incluye la prohibición de presentar:  - Actores o voces de niños; - Personajes animados (animales con licencia o sin licencia, niños, objetos); - Celebridades y deportistas; -Regalos promocionales o incentivos (obsequios, premios, concursos, juegos interactivos); - Referencias a la vida infantil (por ejemplo, escuela, parques de juegos, palabras populares entre los niños, fantasía) | 2 semanas antes y 2 semanas después de la regulación | Canales de TV de Chile |
| Huse et al | 2020 | Restricción de todas las promociones de precios de las bebidas azucaradas, definidas como reducciones temporales de precios, ofertas de compra múltiple y ofertas de compra uno y lleva uno gratis | N/A | Australia |
| Magnus et al | 2009 | Restricción de publicidad de TV de alimentos y bebidas densos en energía y pobres en nutrientes durante los principales horarios de audiencia infantil (5-14 años): por la mañana durante 1 a 2 horas, y por la tarde/noche durante 5 horas (hasta las 21:00 horas). | N/A | Canales de TV de Australia |
| Miller et al | 2016 | Políticas y prácticas de nutrición a nivel distrital, las cuales incluyen: - Educación nutricional; - Campus cerrado (impedimento de salir de la escuela, incluyendo el horario de almuerzo); - Restricción de la venta de bebidas; - Restricción de la distribución de productos promocionales como caramelos, restaurantes de comida rápida o bebidas azucaradas para los estudiantes, como camisetas, gorras y cubiertas de libros;  - Oferta de alternativas saludables, como agua y leche baja en grasa | N/A | Distritos escolares públicos de Estados Unidos |
| Mytton et al | 2020 | Prohibición de la publicidad de alimentos y bebidas altos en grasas, azúcares y sal (HFSS) desde las 05:30 horas hasta las 21:00 horas (de 5:30 AM a 9:00 PM) | N/A | Canales de TV del Reino Unido |
| Pauzé & Potvin Kent | 2021 | Iniciativa voluntaria liderada por la industria alimentaria de la que participan 16 empresas | Cuatro semanas durante cada período analizado | Canales de TV de Canadá (Toronto) |
| Polacsek et al. | 2012 | Prohibición de la publicidad de marcas de alimentos o bebidas en escuelas con excepciones para los alimentos y bebidas que cumplan con los estándares estatales | N/A | Escuelas del Estado de Maine (Estados Unidos) |
| Potvin Kent & Wanless | 2014 | Iniciativa voluntaria liderada por la industria alimentaria: 16 empresas de alimentos y bebidas no saludables se comprometieron a restringir la publicidad a niños menores de 12 años en múltiples plataformas de medios (televisión, radio, prensa e Internet) y a ofrecer opciones más saludables | Cuatro semanas durante cada período analizado | Canales de TV de Canadá (Toronto y Vancouver) |
| Taillie et al | 2020 | Política integral que incluye: - Adopción del etiquetado frontal de advertencias para alimentos y bebidas altos en energía, azúcares, grasas saturadas y sodio; - Restricción de la venta y promoción de productos etiquetados en las escuelas; - Restricción de publicidad dirigida a NNyA de productos etiquetados | 18 meses antes de la implementación y 18 meses después de la implementación | Chile |

NNyA: Niños, niñas y adolescentes

N/A: No aplica

TV: Televisión

**Anexo 4**: Evaluación de la calidad de los estudios

Evaluación de la calidad ensayo clínico aleatorizado (RoB2)^(2)^

| **Autores y año** | **Proceso de randomización** | **Desviaciones de las intervenciones previstas** | **Falta de datos de resultados** | **Medición del resultado** | **Selección del resultado reportado** | **Sesgo general** |
| --- | --- | --- | --- | --- | --- | --- |
| Brimblecombe et al, 2020 | Low risk | Some concerns | Low risk | Some concerns | Low risk | Some concerns |

Evaluación de la calidad de los estudios observacionales

| **Autores y año** | **Diseño del estudio** | **Evaluación** | | | | | | | | | | | | | | **Calidad Global** |
| --- | --- | --- | --- | --- | --- | --- | --- | --- | --- | --- | --- | --- | --- | --- | --- | --- |
|  |  | 1 | 2 | 3 | 4 | 5 | 6 | 7 | 8 | 9 | 10 | 11 | 12 | 13 | 14 |  |
| Miller et al, 2016 | Transversal | Si | Si | Si | Si | Si | No | No | N/A | Si | No | Si | N/A | N/A | Si | Moderada |
| Polacsek et al, 2012 | Transversal | Si | Si | Si | Si | No | No | No | N/A | Si | No | Si | N/A | N/A | No | Baja |
| Pauzé & Potvin Kent, 2021 | Estudio cuasi experimental | Si | Si | No | No | Si | Si | Si | N/A | N/A | Si | N/A | No |  |  | Baja |
| Correa et al, 2020 | Estudio antes y después no controlado | Si | Si | No | Si | Si | Si | Si | N/A | N/A | Si | No | N/A |  |  | Moderada |
| Potvin Kent & Wanless, 2014 | Estudio antes y después no controlado | Si | Si | No | No | Si | Si | Si | N/A | N/A | No | No | No |  |  | Baja |
| Taillie et al, 2020 | Estudio controlado antes y después | Si | Si | Si | Si | Si | Si | Si | N/A | N/A | Si | Si | Si |  |  | Alta |

Preguntas para evaluar los estudios transversales (Quality Assessment Tool for Observational Cohort and Cross-Sectional Studies)^(3)^

1. Was the research question or objective in this paper clearly stated?

2. Was the study population clearly specified and defined?

3. Was the participation rate of eligible persons at least 50%?

4. Were all the subjects selected or recruited from the same or similar populations (including the same time period)? Were inclusion and exclusion criteria for being in the study prespecified and applied uniformly to all participants?

5. Was a sample size justification, power description, or variance and effect estimates provided?

6. For the analyses in this paper, were the exposure(s) of interest measured prior to the outcome(s) being measured?

7. Was the timeframe sufficient so that one could reasonably expect to see an association between exposure and outcome if it existed?

8. For exposures that can vary in amount or level, did the study examine different levels of the exposure as related to the outcome (e.g., categories of exposure, or exposure measured as continuous variable)?

9. Were the exposure measures (independent variables) clearly defined, valid, reliable, and implemented consistently across all study participants?

10. Was the exposure(s) assessed more than once over time?

11. Were the outcome measures (dependent variables) clearly defined, valid, reliable, and implemented consistently across all study participants?

12. Were the outcome assessors blinded to the exposure status of participants?

13. Was loss to follow-up after baseline 20% or less?

14. Were key potential confounding variables measured and adjusted statistically for their impact on the relationship between exposure(s) and outcome(s)?

Preguntas para evaluar la calidad de los estudios antes-después controlados y no controlados y cuasi experimentales (“Quality Assessment Tool for Before-After (Pre-Post) Studies With No Control Group”)^(3)^

1. Was the study question or objective clearly stated?

2. Were eligibility/selection criteria for the study population prespecified and clearly described?

3. Were the participants in the study representative of those who would be eligible for the test/service/intervention in the general or clinical population of interest?

4. Were all eligible participants that met the prespecified entry criteria enrolled?

5. Was the sample size sufficiently large to provide confidence in the findings?

6. Was the test/service/intervention clearly described and delivered consistently across the study population?

7. Were the outcome measures prespecified, clearly defined, valid, reliable, and assessed consistently across all study participants?

8. Were the people assessing the outcomes blinded to the participants' exposures/interventions?

9. Was the loss to follow-up after baseline 20% or less? Were those lost to follow-up accounted for in the analysis?

10. Did the statistical methods examine changes in outcome measures from before to after the intervention? Were statistical tests done that provided p values for the pre-to-post changes?

11. Were outcome measures of interest taken multiple times before the intervention and multiple times after the intervention (i.e., did they use an interrupted time-series design)?

12. If the intervention was conducted at a group level (e.g., a whole hospital, a community, etc.) did the statistical analysis take into account the use of individual-level data to determine effects at the group level?

Evaluación de la calidad de los reportes de impacto económico

| **Autores y año** | **Diseño del estudio** | **Evaluación** | | | | | | | | | | | | | | | | | | | | | | | | | | | | **Calidad Global** |
| --- | --- | --- | --- | --- | --- | --- | --- | --- | --- | --- | --- | --- | --- | --- | --- | --- | --- | --- | --- | --- | --- | --- | --- | --- | --- | --- | --- | --- | --- | --- |
|  |  | 1 | 2 | 3 | 4 | 5 | 6 | 7 | 8 | 9 | 10 | 11 | 12 | 13 | 14 | 15 | 16 | 17 | 18 | 19 | 20 | 21 | 22 | 23 | 24 | 25 | 26 | 27 | 28 |  |
| Brown et al, 2018 | Modelo | Si | Si | Si | Si | Si | Si | Si | Si | Si | Si | Si | Si | Si | Si | Si | Si | Si | Si | Si | Si | No | Si | Si | Si | No | Si | Si | Si | Alta |
| Huse et al, 2019 | Modelo | Si | Si | Si | Si | Si | Si | Si | Si | Si | Si | Si | Si | Si | Si | Si | Si | Si | Si | Si | Si | Si | Si | Si | Si | Si | Si | Si | Si | Alta |
| Magnus et al, 2009 | Modelo | Si | Si | Si | Si | Si | Si | Si | Si | Si | Si | Si | Si | Si | Si | Si | Si | Si | Si | Si | Si | No | Si | Si | Si | No | Si | Si | Si | Alta |
| Mytton et al, 2020 | Modelo | Si | Si | Si | Si | Si | Si | Si | No | Si | Si | Si | Si | Si | Si | Si | Si | Si | Si | Si | Si | No | Si | Si | Si | No | Si | Si | Si | Alta |

CHEERS 2022 checklist^(4)^

| Section/topic | Item no. | Guidance for reporting |
| --- | --- | --- |
| Title | 1 | Identify the study as an economic evaluation and specify the interventions being compared. |
| Abstract | | |
| Abstract | 2 | Provide a structured summary that highlights context, key methods, results, and alternative analyses. |
| Introduction | | |
| Background and objectives | 3 | Give the context for the study, the study question, and its practical relevance for decision making in policy or practice. |
| Methods | | |
| Health economic analysis plan | 4 | Indicate whether a health economic analysis plan was developed and where available. |
| Study population | 5 | Describe characteristics of the study population (such as age range, demographics, socioeconomic, or clinical characteristics). |
| Setting and location | 6 | Provide relevant contextual information that may influence findings. |
| Comparators | 7 | Describe the interventions or strategies being compared and why chosen. |
| Perspective | 8 | State the perspective(s) adopted by the study and why chosen. |
| Time horizon | 9 | State the time horizon for the study and why appropriate. |
| Discount rate | 10 | Report the discount rate(s) and reason chosen. |
| Selection of outcomes | 11 | Describe what outcomes were used as the measure(s) of benefit(s) and harm(s). |
| Measurement of outcomes | 12 | Describe how outcomes used to capture benefit(s) and harm(s) were measured. |
| Valuation of outcomes | 13 | Describe the population and methods used to measure and value outcomes. |
| Measurement and valuation of resources and costs | 14 | Describe how costs were valued. |
| Currency, price date, and conversion | 15 | Report the dates of the estimated resource quantities and unit costs, plus the currency and year of conversion. |
| Rationale and description of model | 16 | If modeling is used, describe in detail and why used. Report if the model is publicly available and where it can be accessed. |
| Analytics and assumptions | 17 | Describe any methods for analysing or statistically transforming data, any extrapolation methods, and approaches for validating any model used. |
| Characterizing heterogeneity | 18 | Describe any methods used for estimating how the results of the study vary for subgroups. |
| Characterizing distributional effects | 19 | Describe how impacts are distributed across different individuals or adjustments made to reflect priority populations. |
| Characterizing uncertainty | 20 | Describe methods to characterize any sources of uncertainty in the analysis. |
| Approach to engagement with patients and others affected by the study | 21 | Describe any approaches to engage patients or service recipients, the general public, communities, or stakeholders (such as clinicians or payers) in the design of the study. |
| Results | | |
| Study parameters | 22 | Report all analytic inputs (such as values, ranges, references) including uncertainty or distributional assumptions. |
| Summary of main results | 23 | Report the mean values for the main categories of costs and outcomes of interest and summarize them in the most appropriate overall measure. |
| Effect of uncertainty | 24 | Describe how uncertainty about analytic judgments, inputs, or projections affect findings. Report the effect of choice of discount rate and time horizon, if applicable. |
| Effect of engagement with patients and others affected by the study | 25 | Report on any difference patient/service recipient, general public, community, or stakeholder involvement made to the approach or findings of the study |
| Discussion | | |
| Study findings, limitations, generalizability, and current knowledge | 26 | Report key findings, limitations, ethical or equity considerations not captured, and how these could affect patients, policy, or practice. |
| Source of funding | 27 | Describe how the study was funded and any role of the funder in the identification, design, conduct, and reporting of the analysis |
| Conflicts of interest | 28 | Report authors conflicts of interest according to journal or International Committee of Medical Journal Editors requirements. |

Referencias

1. Page MJ, Moher D, Bossuyt PM, Boutron I, Hoffmann TC, Mulrow CD, et al. PRISMA 2020 explanation and elaboration: updated guidance and exemplars for reporting systematic reviews. BMJ. 2021;372:n160. doi:10.1136/bmj.n160

2. Sterne JAC, Savović J, Page MJ, Elbers RG, Blencowe NS, Boutron I, et al. RoB 2: a revised tool for assessing risk of bias in randomised trials. BMJ. 2019;366:l4898. doi:10.1136/bmj.l4898

3. Study Quality Assessment Tools [Internet]. Disponible en: https://www.nhlbi.nih.gov/health-topics/study-quality-assessment-tools

4. Husereau D, Drummond M, Augustovski F, de Bekker-Grob E, Briggs AH, Carswell C, et al. Consolidated Health Economic Evaluation Reporting Standards (CHEERS) 2022 Explanation and Elaboration: A Report of the ISPOR CHEERS II Good Practices Task Force. Value Health. 2022;25(1):10–31. doi:10.1016/j.jval.2021.10.008

1. MS: Material suplementario [↑](#footnote-ref-1)
