# Supplementary material for: Impact of policies restricting advertising, promotion, and sponsorship of sugar-sweetened beverages: A systematic review
Source: Rev Peru Med Exp Salud Publica. 2025 Feb 17;42(1):28–36. doi: 10.17843/rpmesp.2025.421.14023 (PMC12176025; doi:10.17843/rpmesp.2025.421.14023)
Supplement: Translation

# Translation: Impacto de políticas de restricción de publicidad, promoción y patrocinio de bebidas azucaradas: Una revisión sistemática

**Authors:** Leila Guarnieri, Lucas Perelli, Marcos Clausen, Germán Guaresti, Natalia Espinola, Andrea Graciano, Andrea Alcaraz

**Affiliations:** 1 Fundación InterAmericana del Corazón Argentina, Buenos Aires, Argentina.; 2 Instituto de Efectividad Clínica y Sanitaria, Departamento de Evaluaciones de Tecnologías Sanitarias, Buenos Aires, Argentina.; 3 Hospital Zonal Dr. Ramón Carrillo, Bariloche, Argentina.; 4 Universidad Nacional de Río Negro, Sede Andina, Bariloche, Argentina.; 5 Universidad de Buenos Aires, Buenos Aires, Argentina.

**Keywords:** Bebidas Azucaradas, Publicidad, Política de Salud, Enfermedades no Transmisibles

## Abstract

**Objetivos.:**

Sintetizar la evidencia sobre el impacto de la implementación de la prohibición de publicidad, promoción y patrocinio (PPP) de bebidas azucaradas (BA) en términos de disminución de consumo, exposición publicitaria y desenlaces clínicos relevantes.

**Materiales y métodos.:**

Revisión sistemática de estudios publicados entre 2001-2021 en las bases de datos PubMed, Embase, Global Health, CINAHL y LILACS escritos en inglés, portugués o español. Se incluyeron estudios experimentales, observacionales y modelos económicos. Se evaluó el riesgo de sesgo mediante RoB2, Quality Assessment Tool for Observational Cohort and Cross-Sectional Studies, Quality Assessment Tool for Before-After (Pre-Post) Studies With No Control Group y Consolidated Health Economic Evaluation Reporting Standards 2022. Se realizó una síntesis descriptiva de los estudios.

**Resultados.:**

Se seleccionaron 11 de 1146 estudios identificados. Debido a la heterogeneidad en los desenlaces no fue posible realizar un meta-análisis. Las intervenciones incluidas correspondieron a una política integral; restricciones de publicidad televisiva, de promociones, en punto de venta y en escuelas. Se hallaron cambios en desenlaces clínicos (obesidad, enfermedad cardiovascular, diabetes, cáncer), de índole económica (compra, venta, costo-efectividad, otros desenlaces económicos), en exposición y en consumo. La mayoría de las medidas de efecto evaluadas registraron disminuciones a partir de las intervenciones. Aún hacen falta más estudios sobre políticas efectivamente implementadas. Los resultados de los trabajos incluidos deben ser interpretados contemplando sus limitaciones metodológicas.

**Conclusiones.:**

Las políticas de restricción de la PPP de las BA pueden resultar efectivas, sobre todo para disminuir su consumo en niños, niñas y adolescentes, impactando positivamente en su salud.

## INTRODUCCIÓN

Las enfermedades no transmisibles (ENT), como las cardiovasculares, la diabetes y ciertos tipos de cáncer, son la principal causa de muerte en el mundo; afectan a todos los grupos de edad y a todos los países ^1^. El porcentaje de muertes atribuibles a ENT aumentó del 60,8% en el año 2000 al 73,6% en 2019 a nivel mundial y, particularmente, del 77,2% al 81,3% en la Región de las Américas ^2^. Las ENT tienen además un importante costo atribuible para los sistemas de salud, así como para la sociedad en general, por lo que esta enorme carga de enfermedad y económica, representa una barrera significativa para alcanzar los Objetivos de Desarrollo Sostenible (ODS) ^3^.

El sobrepeso y la obesidad son factores de riesgo importantes de ENT. A nivel mundial las estadísticas resultan alarmantes tanto en la adultez como en la niñez y adolescencia ^4^. La prevalencia de obesidad en niños, niñas y adolescentes (NNyA) entre 5 y 19 años de todo el mundo se incrementó de 2,9% (IC95% de 2,6 a 3,2) en el año 2000 al 4,9% (IC95% de 4,6 a 5,3) en 2010 frente al 6,8% (IC95% de 6,1 a 7,6) en 2016. En la población adulta también se han registrado incrementos en las cifras de obesidad en las últimas décadas, alcanzando en el 2016 al 13,1% (IC95% de 12,4 a 13,9) de este grupo etario ^2^.

El incremento en los niveles de sobrepeso y obesidad se ha relacionado con cambios en los patrones de consumo de alimentos, como consecuencia de estímulos que favorecen el consumo de productos de alto nivel calórico y bajo valor nutricional ^5^. En los entornos alimentarios, el marketing y la publicidad influyen tanto en las preferencias, como en las decisiones de compra y consumo de alimentos, especialmente en NNyA ^6^^,^^7^. La publicidad y otras formas de mercadotecnia de alimentos y bebidas dirigidas a NNyA tienen un gran alcance y se centran principalmente en productos con contenido excesivo en grasas, azúcares o sodio, como es el caso de las BA ^8^. La evidencia muestra que los niños y las niñas hasta los once años no poseen la madurez necesaria para diferenciar el contenido publicitario de otro tipo de mensajes ^9^, aspecto que vuelve a esta población especialmente vulnerable. Por eso, los organismos internacionales como OPS/OMS y UNICEF llaman a los Estados a tomar acciones inminentes e inmediatas capaces de abordar los múltiples matices de esta problemática, como la regulación de la publicidad, promoción y patrocinio (PPP) de los productos no saludables ^5^^,^^10^^-^^12^.

El consumo de BA constituye una fuente importante de calorías, generalmente sin aportar otros nutrientes más que azúcares, aumentando el riesgo de padecer obesidad, diabetes, enfermedades cardíacas, cerebrovasculares, osteomusculares, insuficiencia renal, demencia, asma, diversos tipos de cáncer y caries ^13^^,^^14^. Además, tanto la obesidad como las caries pueden derivar en otras problemáticas sociales como discriminación o falta de oportunidades laborales ^15^.

En este marco, la restricción de la PPP de productos alimenticios y bebidas no saludables, en especial la que se dirige a NNyA, intenta reducir el consumo de dichos productos y es una medida costo-efectiva, viable y en general aceptada por los gobiernos, los decisores políticos y la población ^7^. Contar con evidencia sistematizada acerca del impacto en la salud de la implementación de este tipo de regulaciones resulta esencial para promover políticas efectivas. Dado que aún no existen estudios que se hayan centrado específicamente en las medidas de restricción de la publicidad de BA, en este estudio se realizó una búsqueda sistemática con el objetivo de resumir la evidencia sobre el impacto de la implementación de la prohibición de la PPP de las BA en términos de disminución de consumo, exposición publicitaria y desenlaces clínicos relevantes.

**MENSAJE CLAVE**

Motivación para el estudio. La restricción de la publicidad, promoción y patrocinio (PPP) de productos alimenticios y bebidas no saludables intenta reducir su consumo y proteger la salud poblacional. Aún no existen revisiones que hayan evaluado el impacto de restringir la PPP de bebidas azucaradas (BA).

Principales hallazgos. Mediante una revisión sistemática se encontró que las políticas integrales que incluyen restricciones publicitarias en TV, así como limitaciones en puntos de venta y escuelas, pueden reducir efectivamente el consumo de BA, especialmente entre niños y adolescentes, disminuir la obesidad, enfermedades cardiovasculares, diabetes y cáncer, y generar beneficios económicos.

Implicancias. Contar con evidencia sobre el impacto en la salud de este tipo de intervenciones resulta esencial para promover medidas efectivas.

## MATERIALES Y MÉTODOS

### Diseño de estudio

Se realizó una revisión sistemática de la literatura publicada siguiendo las directrices de PRISMA ^16^. (Material suplementario, anexo 1) 

### Estrategia de búsqueda

Se realizó una búsqueda de estudios realizados a nivel nacional, regional e internacional; indexados en los buscadores bibliográficos PubMed, Embase, Global Health, CINAHL y LILACS (Latin American and Caribbean Health Science Literature). La estrategia de búsqueda completa se encuentra en el material suplementario (Anexo 2).

### Criterios de selección

Los criterios de inclusión fueron estudios publicados durante los últimos 20 años (2001-2021); en idioma inglés, portugués o español; que describieran políticas que regulen la PPP de BA, obligatorias o voluntarias, de forma independiente o complementaria a otras políticas; estudios que midan el impacto en el consumo, compras, ventas, intención de compra, exposición y/o desenlaces clínicos (obesidad, caries, enfermedad cardiovascular, hipertensión arterial, dislipemias, insulinorresistencia, diabetes, cáncer y otros desenlaces clínicos relacionados); y estudios con diseños experimentales, estudios controlados antes y después, estudios antes y después no controlados, estudios cuasi-experimentales, estudios transversales, modelos económicos, evaluaciones económicas y estudios de costos. Se excluyeron los estudios que no cumplieron con los criterios de inclusión y aquellos que analizaron el impacto de las políticas de regulación de la PPP de bebidas endulzadas no azucaradas.

### Selección de estudios y recolección de datos

Cada uno de los estudios identificados fue evaluado por dos revisores del equipo investigador asignados aleatoriamente en cada etapa del proceso, quienes inicialmente por el título y el resumen, y posteriormente, a través de la lectura de los textos completos, seleccionaron aquellos que cumplían los criterios de inclusión utilizando el programa informático COVIDENCE® ^17^. Cuando surgieron discrepancias entre los revisores, se debatieron entre el equipo investigador completo para arribar a una decisión final consensuada. Luego, cada uno de los estudios seleccionados fue asignado aleatoriamente a un investigador para realizar la identificación y extracción de la información relevante; resolviendo grupalmente las inquietudes individuales que surgieron durante el proceso para arribar a una conclusión.

Se realizó una síntesis descriptiva de las principales características de los estudios considerando el tipo de diseño, las intervenciones, su grado de implementación y los desenlaces evaluados. No fue posible realizar un metaanálisis dada la elevada heterogeneidad de las intervenciones, las poblaciones objetivo y los desenlaces evaluados en cada estudio.

### Evaluación del riesgo de sesgo de los estudios incluidos (calidad)

Pares de revisores evaluaron de forma independiente el riesgo de sesgo (calidad) de los estudios incluidos. En caso de desacuerdo, se resolvió por consenso grupal. Debido a la naturaleza de la pregunta de investigación, no se consideró aplicable una única herramienta, sino que se utilizó una combinación de instrumentos validados según los diseños de los estudios incluidos. Para los ensayos clínicos aleatorizados se utilizó la herramienta RoB2 ^18^; para los diseños transversales la herramienta Quality Assessment Tool for Observational Cohort and Cross-Sectional Studies ^19^; para los estudios antes-después controlados y no controlados y para los cuasi experimentales se usó la herramienta Quality Assessment Tool for Before-After (Pre-Post) Studies With No Control Group ^19^; y para los modelos económicos se utilizó la lista de verificación Consolidated Health Economic Evaluation Reporting Standards 2022 ^20^, desarrollada con el propósito principal de servir de guía para los reportes.

## RESULTADOS

### Selección de estudios

Se identificaron 1146 estudios desde las bases de datos con la estrategia de búsqueda. Después de remover por duplicados (n=311), los 835 restantes fueron analizados aplicando los criterios de inclusión por título y resumen. Un total de 758 estudios fueron excluidos en esta primera etapa y los 77 restantes fueron identificados como elegibles para el análisis por texto completo. Finalmente, 11 artículos ^21^^-^^31^ fueron elegibles para esta revisión sistemática (Tabla 1). El proceso completo de selección de estudios se muestra en la Figura 1.

**Tabla 1:** Descripción de los estudios incluidos.

Autores	Año	País	Diseño	Intervención evaluada	Variables analizadas									
					Obesidad	Enfermedad cardiovascular	Diabetes	Cáncer	Compra	Venta	Costo-efectividad	Otros desenlaces económicos	Consumo	Exposición
Brimblecombe *et al*	2020	Australia	Ensayo clínico aleatorizado	Restricciones en PDV						x				
Brown *et al*	2018	Australia	Modelo económico	Restricción de publicidad en TV dirigida a NNyA	x						x		x	
Correa *et al*	2020	Chile	Estudio antes y después no controlado	Restricción de la publicidad en TV dirigida a NNyA										x
Huse *et al*	2020	Australia	Modelo	Restricción de promociones	x	x	x	x			x	x	x	
Magnus *et al*	2009	Australia	Modelo	Restricción de la publicidad en TV	x						x			
Miller *et al*	2016	Estados Unidos	Transversal	Restricción de promociones en escuelas									x	
Mytton *et al*	2020	Reino Unido	Modelo	Restricción de la publicidad en TV	x						x	x	x	x
Pauzé & Potvin Kent	2021	Canadá	Estudio cuasi experimental	Restricción de la publicidad en TV dirigida a NNyA (autorregulación)										x
Polacsek *et al*	2012	Estados Unidos	Transversal	Restricción de la publicidad en escuelas										x
Potvin Kent & Wanless	2014	Canadá	Estudio antes y después no controlado	Restricción de la publicidad en TV dirigida a NNyA (autorregulación)										x
Taillie *et al*	2020	Chile	Estudio controlado antes y después	Política integral ^a^					x					

PDV: Punto de venta; NNyA: Niños, niñas y adolescentes; TV: Televisión; ^ª^Incluye etiquetado frontal, restricción de la publicidad dirigida a NNyA y restricción de la venta en las escuelas

**Figura 1:** Proceso de selección de los estudios incluidos en la revisión

### Características generales

En cuanto a los diseños metodológicos se encontró gran heterogeneidad entre los estudios, resultando la mayoría modelos de impacto económico. En relación a las intervenciones, de los once artículos, seis analizaron restricciones de publicidad en televisión ^22^^,^^23^^,^^25^^,^^27^^,^^28^^,^^30^, dos restricciones en escuelas ^26^^,^^29^, uno restricciones de promociones (entendidas como las reducciones temporales de los precios y ofertas de productos) ^24^, uno restricciones en el punto de venta ^21^^)^ y uno sobre política integral ^31^ (Tabla 1). En el material suplementario (Anexo 3) se presentan más detalles sobre las características de cada una de las intervenciones evaluadas en los estudios.

La mayoría de los estudios evaluaron la exposición (n=5) ^23^^,^^27^^-^^30^ seguido de obesidad (n=4) ^22^^,^^24^^,^^25^^,^^27^, costo-efectividad (n=4) ^22^^,^^24^^,^^25^^,^^27^, consumo (n=4) ^22^^,^^24^^,^^26^^,^^27^, otros desenlaces económicos (n=2) ^24^^,^^27^, enfermedad cardiovascular (n=1) ^24^, diabetes (n=1) ^24^, cáncer (n=1) ^24^, compra (n=1) ^31^^)^ y venta (n=1) ^21^. Ningún trabajo evaluó el impacto en relación a caries, hipertensión arterial, dislipidemias e insulinorresistencia (Tabla 1).

### Evaluación de la calidad de la evidencia (riesgo de sesgo)

En relación a la calidad de la evidencia, en cinco de los estudios la misma fue calificada como alta ^22^^,^^24^^,^^25^^,^^27^^,^^31^, en tres como moderada ^21^^,^^23^^,^^26^ y en otros tres como baja ^28^^-^^30^. (Tabla 2). En el material suplementario (Anexo 4) se presentan las tablas correspondientes a la evaluación de la calidad de los estudios según cada diseño.

**Tabla 2:** Calidad de los estudios incluidos en la revisión sistemática.

Autor y año	Diseño del estudio	Herramienta utilizada	Resultado
Brimblecombe *et al*, 2020	Ensayo clínico aleatorizado	RoB2	Moderada
Brown *et al*, 2018	Modelo económico	CHEERS 2022	Alta
Correa *et al*, 2020	Estudio antes y después no controlado	Quality Assessment Tool for Before-After (Pre-Post) Studies With No Control Group (NIH)	Moderada
Huse *et al*, 2019	Modelo	CHEERS 2022	Alta
Magnus *et al,* 2009	Modelo	CHEERS 2022	Alta
Miller *et al*, 2016	Transversal	Quality Assessment Tool for Observational Cohort and Cross-Sectional Studies (NIH)	Moderada
Mytton *et al*, 2020	Modelo	CHEERS 2022	Alta
Pauzé & Potvin Kent, 2021	Estudio cuasi experimental	Quality Assessment Tool for Before-After (Pre-Post) Studies With No Control Group (NIH)	Baja
Polacsek *et al*, 2012	Transversal	Quality Assessment Tool for Observational Cohort and Cross-Sectional Studies (NIH)	Baja
Potvin Kent and Wanless, 2014	Estudio antes y después no controlado	Quality Assessment Tool for Before-After (Pre-Post) Studies With No Control Group (NIH)	Baja
Taillie *et al*, 2020	Estudio controlado antes y después	Quality Assessment Tool for Before-After (Pre-Post) Studies With No Control Group (NIH)	Alta

CHEERS: Consolidated Health Economic Evaluation Reporting Standards.

### Resultados según intervenciones

En relación al estudio que analizó el impacto de una política integral ^31^ luego de 18 meses de implementada en Chile, la cual incluye la adopción del etiquetado frontal de advertencias, restricción de la publicidad dirigida a NNyA de productos etiquetados, y prohibición de su venta y publicidad en las escuelas, se evidenció una reducción en las compras de las bebidas etiquetadas con las advertencias correspondiente a -23,7% (-23,8% a -23,7%).

En cuanto a las restricciones de publicidad en televisión, al evaluar la exposición, los diversos estudios no resultaron concluyentes: mientras un modelo de impacto económico ^27^ y otro trabajo sobre la política implementada en Chile ^24^ arrojaron disminuciones, los análisis de la medida voluntaria de la industria de alimentos y bebidas canadiense registraron, en un caso aumentos ^28^ y en otro aumentos y disminuciones ^30^. Respecto a la obesidad, tres trabajos, donde se evaluaron cambios en el IMC ^22^^,^^25^ y en el número de NNyA con obesidad ^(^^27^, hallaron disminuciones en dichas medidas de efecto. Asimismo, en tres modelos ^22^^,^^25^^,^^27^^)^ se demostró que las limitaciones horarias a la publicidad de productos no saludables resultarían costo-efectivas. En dos de los trabajos ^22^^,^^27^ se encontró que las restricciones publicitarias provocarían disminuciones en la ingesta diaria de energía de NNyA. Por su parte, en uno de los estudios se registraron ahorros en costos atribuibles a la aplicación de las restricciones ^27^.

En lo relativo a las restricciones de promociones, un mismo estudio ^24^ reportó disminuciones en el peso corporal y en el IMC de la población, así como nuevos casos evitados y años de vida salvados por enfermedades cardiovasculares, diabetes y cáncer como consecuencia de eliminar todas las promociones de precios de las BA. El mismo trabajo ^24^^)^ también demostró que la intervención resultaría costo-efectiva, arrojaría compensaciones de costos y reduciría la ingesta diaria de energía.

Por su parte, al analizar las restricciones en el punto de venta, un ensayo clínico aleatorizado ^21^ realizado durante 12 semanas demostró que a partir de la implementación de una serie de medidas en tiendas minoristas de localidades australianas, las ventas de azúcares libres de las BA se redujeron en un 6,8% (IC 95%: -10,9% a -2,6%).

Por último, los estudios de restricciones en escuelas realizados en Estados Unidos, arrojaron, en uno de los casos ^26^, que los distritos escolares que aplican restricciones sobre las BA tienen un menor consumo regular de gaseosas; mientras que en otro trabajo se puso en evidencia que, a pesar de encontrarse prohibida la presencia de marcas de productos no saludables por parte del Estado, aún existe una elevada cantidad de estrategias publicitarias en el entorno escolar, muchas de ellas de BA ^29^.

Ninguno de todos los estudios incluidos analizó restricciones globales de la PPP que alcance exclusivamente a las BA (Tabla 3, anexo 3).

**Tabla 3:** Impacto identificado según tipos de intervenciones evaluadas.

Intervención evaluada	Desenlace	Autores y año	Principales resultados
Política integral ^a^	Compra	Taillie *et al*, 2020	El volumen diario promedio per cápita (mL) de bebidas azucaradas "altas en" comprado registró una disminución de 23,7% (−23,8% a −23,7%)
Restricciones de publicidad en TV	Obesidad	Brown *et al*, 2018	El IMC promedio en NNyA de 5 a 15 años (kg/m^2^) registró una disminución de 0,352
		Mytton *et al*, 2020	A partir de la intervención, el número de niños/as de 5 a 17 años con obesidad podría disminuir un 4,6% (IC 95%:1,4%-9,5%), lo que equivale a 40 000 niños/as menos con obesidad
		Magnus *et al*, 2009	A partir de las restricciones se produciría una reducción del IMC (unidades) de 0,04 (IC del 95%: 0,01, 0,08).
	Costo-efectividad	Mytton *et al*, 2020	Las restricciones de publicidad de alimentos y bebidas altos en grasas, azúcares y sal evitarían 240 000 (IC del 95%: 65 000-530 000) DALYs, resultando en un beneficio monetario de £7 400 millones (IC95% £2 0000 -£16 000)
		Magnus *et al*, 2009	La costo-efectividad de la intervención, medida como ICER, resultó en AUD$ 3,70 (IC 95%: $2,40-$7,70) por DALY La intervención se consideró "dominante", por resultar en una ganancia en salud y una reducción en costos
		Brown *et al*, 2018	La costo-efectividad de la intervención, medida como ICER, mostró un 100% de probabilidad de ser "dominante", resultando en 88 396 HALYs salvados (IC del 95%: 54 559-123 199) y un ahorro total de costos de AUD$ 777,9M (IC del 95%: AUD$ 369,8M-AUD$ 1,2B) a nivel poblacional a lo largo de la vida
	Otros desenlaces económicos	Mytton *et al*, 2020	Como consecuencia de la intervención se produciría un: Ahorro de costos sanitarios (millones) de £84 (£23-£190) Ahorro de costos de la asistencia social (millones): £210 (£56-£490) Beneficio monetario neto relacionado a la salud (millones): £7 400 (£2 000-£16 000)
	Exposición	Mytton *et al*, 2020	A partir de la intervención se produciría una reducción media en la exposición (número de anuncios de productos altos en azúcares, grasas y/o sodio vistos por día) en NNyA de 1,5
		Correa *et al*, 2020	Como resultado de la implementación de la política se obtuvo una disminución en los anuncios de TV del: Número de apariciones de "gaseosas": 51% (p < 0,01) Número de apariciones de "bebidas deportivas y energizantes": 23% (p < 0,01)
		Pauzé & Potvin Kent, 2021	La exposición de NNyA a publicidades de bebidas azucaradas (medida como el número de anuncios publicitarios vistos por NNyA) tuvo un incremento entre mayo de 2011 y mayo de 2019 del 495%
		Potvin Kent & Wanless, 2014	La exposición, medida como el número promedio de anuncios de bebidas azucaradas vistos por niños de 2-11 años, mostró diferencias entre 2006 y 2009: en jugos disminuyó un 62,6% en Toronto y un 51,6% en Vancouver; en gaseosas disminuyó un 37,8% en Toronto y aumentó un 11,1% en Vancouver
	Consumo	Mytton *et al*, 2020	Como respuesta a las restricciones, se produciría una reducción en la ingesta media diaria de energía en NNyA de 9,1 kcal/día (IC 95%: 0,5-17,7)
		Brown *et al*, 2018	La implementación de la restricción resultaría en una disminución media en la ingesta de energía en NNyA de 5 a 15 años de 115 kj/día (27,5 kcal)
Restricción de promociones	Obesidad	Huse *et al*, 2019	La intervención arrojó un cambio medio en el peso corporal en la población (kg) de −0,11 (IC 95%: −0,14 a −0,08) y un cambio medio en el IMC de la población (kg/m2): −0,04 (IC 95%: −0,05 a −0,03)
	Enfermedad cardiovascular	Huse *et al*, 2019	Como resultado de la intervención se evitarían 3 609 (IC 95%: 2625-4688) nuevos casos de enfermedad cardíaca y como consecuencia se salvarían 11 941(IC 95%: 8 967-15 322) años de vida
	Diabetes	Huse *et al*, 2019	Como resultado de la intervención se evitarían 14 319 (IC 95%: 10 198-19 282) nuevos casos de diabetes y como consecuencia se salvarían 5 041 (IC 95%: 3 604-6 779) años de vida
	Cáncer	Huse *et al*, 2019	Como resultado de la intervención se evitarían 846 (IC 95%: 395-1485) casos de cáncer colorrectal, mama, endometrial y de riñón y como consecuencia de esto se salvarían 2 798 (IC 95%: 1 822-4 067) años de vida
	Costo-efectividad	Huse *et al*, 2019	La costo-efectividad de la intervención, medida como ICER, resultó ser dominante, con un total de HALYs ganados de 34 260 (24 922-45 504) y un ahorro total de costos de AUD$ 358, 9M (IC del 95%: −AUD$ 260,1M a −AUD$ 477,7 M)
	Otros desenlaces económicos	Huse *et al*, 2019	La intervención produciría compensaciones de costos totales: −AUD376,0M (IC 95%:−AUD277,4M a −AUD494,3M)
	Consumo	Huse *et al,* 2019	La intervención resultó en un cambio medio en la ingesta diaria de energía de −12,52 kJ (IC 95%: −15,91 a −9,58) por persona
Restricciones en PDV	Venta	Brimblecombe *et al*, 2020	La implementación de las restricciones arrojó una reducción en las ventas de azúcares libres de BA (g/total MJ) estadísticamente significativa: -6,8% (-10,9 a -2,6)
Restricciones en escuelas	Consumo	Miller *et al*, 2016	Los distritos que aplican restricciones de productos con promociones tienen un 16% menos de consumo regular de gaseosas
	Exposición	Polacsek *et al*, 2012	Se encontró un promedio de 49 posters y letreros de alimentos y bebidas en diferentes áreas, 45% de la publicidad de máquinas expendedoras fueron de Coca-Cola® y PepsiCo®

NNyA: Niños, niñas y adolescentes; DALYs: Años de vida ajustados por discapacidad; ICER: Relación costo-efectividad incremental; HALYS: Total de años de vida ajustados por salud; PDV: Punto de venta; BA: Bebidas Azucaradas; ^a^ Incluye etiquetado frontal, restricción de la publicidad dirigida a NNyA y restricción de la venta en las escuelas. £: Libra esterlina.

## DISCUSIÓN

Los resultados de este trabajo muestra que las intervenciones de restricción de la PPP de productos alimenticios y bebidas con cantidades excesivas de azúcares, grasas y/o sodio, como las BA podrían ser beneficiosas, en cuanto a sus desenlaces clínicos, económicos, de consumo y exposición. Estos resultados son consistentes con lo observado por Boyland *et al*^32^ quienes encontraron que las restricciones del marketing de productos no saludables pueden ayudar a disminuir la compra de los mismos, sus consecuencias negativas para la salud, y también, a limitar la exposición y/o el poder de dicho marketing.

Uno de nuestros principales hallazgos evidencia que, al implementar una política integral de etiquetado frontal de advertencias, restricción de la publicidad dirigida a NNyA de los productos etiquetados, y la prohibición de su venta y publicidad en las escuelas en Chile, se registraron disminuciones marcadas en la compra de BA, resultando una medida efectiva para mejorar la salud ^31^. Hay que tener en cuenta que en Chile las bebidas endulzadas con edulcorantes no calóricos no se encuentran alcanzadas por las restricciones y las disminuciones en las compras de BA se acompañaron de un aumento en las de bebidas con edulcorantes ^33^. En este sentido, resultará fundamental monitorear el impacto de políticas similares adoptadas en países como México ^34^^)^ y Argentina ^35^ donde los productos con edulcorantes no calóricos sí se encuentran alcanzados.

Además, es de destacar que a nivel mundial la mayoría de las políticas implementadas corresponden a restricciones de publicidad televisiva dirigida a NNyA ^36^; concordantemente, la mayoría de los estudios incluidos en este trabajo son de restricciones en televisión, y en ellos se da cuenta especialmente del impacto de estas medidas en la exposición a la publicidad de productos no saludables. Sin embargo, dado que cada vez es mayor la exposición de los NNyA a la publicidad efectuada en videojuegos o en redes sociales como Youtube, Tik Tok, e Instagram ^37^, los organismos internacionales recomiendan que las regulaciones incluyan también a los medios digitales ^38^. Al respecto, si bien algunos países del mundo, como Argentina, Chile y el Reino Unido han adoptado medidas que incorporan estos canales ^35^^,^^36^, su monitoreo y fiscalización por parte de los gobiernos se reconoce como uno de los principales desafíos a sortear ^38^.

Otro resultado que merece ser destacado es la falta de efectividad de las iniciativas voluntarias de la industria de alimentos y bebidas para disminuir la exposición de los NNyA a la publicidad de BA, ya que, contrariamente al objetivo que deberían perseguir, dos evaluaciones incluidas en este estudio muestran que desde su implementación se produjeron incrementos en sus indicadores ^28^^,^^30^. Estos resultados coinciden con lo observado por Théodore *et al*, quienes demostraron que, a pesar de la iniciativa de autorregulación en México, las empresas de BA continuaban implementando estrategias publicitarias para llegar a la audiencia infantil ^39^. En relación a esto, es importante resaltar que la Organización Mundial de la Salud recomienda que las políticas destinadas a restringir la publicidad de alimentos y bebidas no saludables dirigida a NNyA sean obligatorias ^7^.

Además, este trabajo evidencia que, aun cuando se trate de restricciones focalizadas en el punto de venta, donde se incluyan estrategias como la ausencia de actividad promocional y de alimentos y bebidas discrecionales disponibles en el mostrador ^21^, se pueden obtener resultados que contribuyan a mejorar la salud. En este sentido, la Organización Mundial de la Salud ha reconocido a los comercios minoristas como un eslabón central del entorno alimentario, destacando la importancia de las intervenciones sobre estos ámbitos ^40^, por lo que las restricciones en los puntos de venta adaptadas a cada contexto en particular, también podrían ser estrategias efectivas.

 En cuanto a las limitaciones, cabe destacar que una parte importante de los estudios incluidos corresponden a modelos de impacto económico ^22^^,^^24^^,^^25^^,^^27^, por lo cual el impacto real de la implementación de las intervenciones analizadas, podrían presentar diferencias respecto a lo reportado. Además, varios trabajos resultaron ser de calidad baja ^28^^-^^30^, por lo que sus resultados merecen ser interpretados con cautela. A su vez, el estudio cuasiexperimental ^28^ y los antes y después no controlados ^23^^,^^30^ podrían tener sesgos especialmente relativos a la selección de las muestras en los períodos analizados. Además, debido a la heterogeneidad de los resultados hallados en los diversos estudios no fue posible agrupar los mismos, dificultando su comparación.

Por otra parte, como limitación se destacan los vacíos de evidencia sobre políticas implementadas de restricción global de la PPP de las BA. También es preciso señalar la escasez de estudios que den cuenta del impacto en relación a desenlaces clínicos, como diabetes y enfermedad cardiovascular. A pesar de esto, los modelos de impacto económico incluidos en esta revisión, otorgan evidencia sobre la potencial efectividad de estas medidas, contribuyendo a saldar dichos vacíos. Además, en algunos estudios ^22^^,^^27^, se reportan desenlaces por restricciones de PPP de productos altos en azúcares, grasas y sal, sin centrarse exclusivamente en las BA. De todas maneras, dado que las BA se encuentran entre los principales productos ultraprocesados publicitados ^41^^)^ y que aportan la mitad de los azúcares de la dieta ^42^, podría atribuirse a las restricciones sobre éstas una gran parte del impacto de las medidas que restringen la PPP de productos altos en nutrientes críticos en general.

La principal fortaleza de este trabajo radica en que es una de las pocas revisiones sistemáticas disponibles sobre el impacto de restricciones de PPP de productos alimenticios y bebidas no saludables, como son las BA. Los resultados de este estudio dan cuenta de la efectividad de distintas intervenciones a través de diferentes variables y podrían ser utilizados para apoyar la promoción de políticas efectivas que contribuyan a mejorar la salud de las personas, con especial énfasis en NNyA.

En conclusión, este estudio evidencia que las políticas que incluyen una restricción de la PPP de las BA podrían resultar efectivas para disminuir su consumo, lo cual podría derivar en un beneficio para la salud, especialmente de NNyA. Sin embargo, es necesario continuar trabajando en evaluaciones del impacto a mediano y largo plazo de las medidas que están comenzando a ser implementadas en distintos países del mundo, para poder contar con información acerca de su efectividad en el consumo y en indicadores clínicos, como obesidad, enfermedad cardiovascular, diabetes, y cáncer. Además, resultará necesario avanzar en investigaciones futuras que evidencien el impacto de las políticas de restricción de PPP de BA que alcanzan a los medios digitales, así como también en la aplicación de metodologías que ayuden a realizar un adecuado monitoreo de los mismos.
